# Benefits, harms and cost-effectiveness of cervical screening, triage and treatment strategies for women in the general population

**DOI:** 10.1038/s41591-023-02600-4

**Published:** 2023-12-12

**Authors:** Kate T. Simms, Adam Keane, Diep Thi Ngoc Nguyen, Michael Caruana, Michaela T. Hall, Gigi Lui, Cindy Gauvreau, Owen Demke, Marc Arbyn, Partha Basu, Nicolas Wentzensen, Beatrice Lauby-Secretan, Andre Ilbawi, Raymond Hutubessy, Maribel Almonte, Silvia De Sanjosé, Helen Kelly, Shona Dalal, Linda O. Eckert, Nancy Santesso, Nathalie Broutet, Karen Canfell

**Affiliations:** 1 The Daffodil Centre, University of Sydney, a joint venture with Cancer Council NSW, Sydney, NSW Australia; 2 Child Health Evaluative Sciences, The Hospital for Sick Children Research Institute, Toronto, ON Canada; 3 SUCCESS Project, Expertise France, Paris, France; 4 Global Diagnostics, Clinton Health Access Initiative, Kigali, Rwanda; 5 Unit of Cancer Epidemiology, Belgian Cancer Centre, Sciensano, Brussels, Belgium; 6 Department of Human Structure and Repair, Faculty of Medicine and Health Sciences, University Ghent, Ghent, Belgium; 7 Early Detection, Prevention and Infections Branch, International Agency for Research on Cancer, Lyon, France; 8 Division of Cancer Epidemiology and Genetics, National Cancer Institute, National Institutes of Health, Rockville, MD USA; 9 Evidence Synthesis and Classification Branch, International Agency for Research on Cancer (IARC), Lyon, France; 10 Department for the Management of Noncommunicable Diseases, Disability, Violence and Injury Prevention, World Health Organization, Geneva, Switzerland; 11 Department of Immunization, Vaccines and Biologicals, World Health Organization, Geneva, Switzerland; 12 Department of Sexual and Reproductive Health, World Health Organization, Geneva, Switzerland; 13 ISGlobal, Barcelona, Spain; 14 London School of Hygiene & Tropical Medicine, London, UK; 15 Department of Global HIV, Hepatitis and Sexually Transmitted Infections Programmes, World Health Organization, Geneva, Switzerland; 16 Department of Global Health, University of Washington, Seattle, WA USA; 17 Department of Obstetrics and Gynecology, University of Washington, Seattle, WA USA; 18 Department of Health Research Methods, Evidence, and Impact, McMaster University, Hamilton, ON Canada; 19 Department of Reproductive Health and Research, World Health Organization, Geneva, Switzerland

**Keywords:** Epidemiology, Health policy, Health care economics

## Abstract

In 2020, the World Health Organization (WHO) launched a strategy to eliminate cervical cancer as a public health problem. To support the strategy, the WHO published updated cervical screening guidelines in 2021. To inform this update, we used an established modeling platform, *Policy1-Cervix*, to evaluate the impact of seven primary screening scenarios across 78 low- and lower-middle-income countries (LMICs) for the general population of women. Assuming 70% coverage, we found that primary human papillomavirus (HPV) screening approaches were the most effective and cost-effective, reducing cervical cancer age-standardized mortality rates by 63–67% when offered every 5 years. Strategies involving triaging women before treatment (with 16/18 genotyping, cytology, visual inspection with acetic acid (VIA) or colposcopy) had close-to-similar effectiveness to HPV screening without triage and fewer pre-cancer treatments. Screening with VIA or cytology every 3 years was less effective and less cost-effective than HPV screening every 5 years. Furthermore, VIA generated more than double the number of pre-cancer treatments compared to HPV. In conclusion, primary HPV screening is the most effective, cost-effective and efficient cervical screening option in LMICs. These findings have directly informed WHO’s updated cervical screening guidelines for the general population of women, which recommend primary HPV screening in a screen-and-treat or screen-triage-and-treat approach, starting from age 30 years with screening every 5 years or 10 years.

## Abstract (web summary)

A modelled evaluation of screening and treatment strategies for prevention of cervical cancer in 78 low- and lower-middle-income countries provides evidence to support the World Health Organization’s recommendation of primary HPV testing for women in the general population.

## Main

In 2020, an estimated 604,000 women were diagnosed with cervical cancer, and 342,000 women died from the disease, with 47% of these deaths occurring in low- and lower-middle-income countries (LMICs) and a further 40% in upper-middle-income countries. Longstanding issues with limited access to cervical cancer prevention and cancer treatment services in LMICs means that, on average, age-standardized cervical cancer mortality rates are more than four times higher than in high-income countries^1^.

In May 2018, the Director-General of the World Health Organization (WHO) issued a call to action to eliminate cervical cancer as a public health problem^2^. A global strategy was requested and then endorsed by Member States, and, in November 2020, the strategy was launched^3^. The strategy recommends that countries implement the ‘90–70–90’ intervention targets by 2030, which are: (1) 90% of girls fully vaccinated with the human papillomavirus (HPV) vaccine by 15 years of age; (2) 70% of women screened using a high-performance test by 35 years of age and again by 45 years of age; and (3) 90% of women identified with cervical pre-cancer or invasive cervical cancer have access to adequate treatment and care^3^. Countries will subsequently be considered to have eliminated cervical cancer as a public health problem when rates of new cases fall below 4 per 100,000 women-years. Modeling performed by the WHO Cervical Cancer Elimination Modelling Consortium (CCEMC) found that, if the 2030 triple-intervention targets are achieved in 78 LMICs, cervical cancer could be eliminated in all LMICs and a total of 74.1 million cancer cases and 62.6 million deaths would be averted over the course of the century^4,5^.

In 2013, the WHO published a Comprehensive Cervical Cancer Control manual, which included screening recommendations for women with or without HIV^6^. For women aged 30–49 years with negative or unknown HIV status, primary HPV screening was recommended at least every 5 years in settings with adequate resources to implement the test. In settings without adequate resources for primary HPV screening, primary visual inspection with acetic acid (VIA) at 3–5-yearly intervals was recommended. For all settings, either ablation or large loop excision of the transformation zone (LLETZ) was recommended for women requiring pre-cancer treatment. For women with HIV^+^ status or unknown status in areas with high endemic HIV infection, the WHO recommended that screening intervals should be no longer than 3 years.

Updated evidence on HPV screening and evidence to support using ablative treatment is now available. Many high-income countries are transitioning from primary cytology to primary HPV testing based on evidence that primary HPV is a more effective and cost-effective primary screening approach^7,8^. Several VIA-based screening experiences have been documented in LMICs. A large community-based randomized controlled trial in India, following more than 70,000 women after multiple rounds of VIA screening over 12 years, found that there was no significant reduction in cervical cancer incidence in women who were screened with VIA compared to unscreened women (relative risk (RR) = 0.97; (95% confidence interval (CI): 0.80–1.19)), but a mortality reduction of 31% (RR = 0.69; (95% CI: 0.54–0.88))^9^, implying some cancer downstaging but low sensitivity for detecting high-grade lesions that could progress to cancer. An earlier study found no significant reduction in incidence of stage II+ cancer and mortality after one round of VIA screening offered to more than 30,000 women^10^. An analysis of studies performed in women living with HIV found that, in studies where more than 95% of women had histological verification of disease, VIA had a sensitivity to detect CIN2+ of 56% (95% CI: 45.4–66.1%)^11^. The 2022 release of a new Handbook of Cervical Screening by the International Agency on Research on Cancer (IARC) synthesized the updated evidence on primary screening technologies, concluding that, although several methods currently used in screening are effective in reducing the incidence of and the mortality associated with cervical cancer, HPV testing alone is the most effective given its balance of benefits and harms^12^.

In response to these developments, the WHO initiated the development of updated cervical screening and treatment guidelines in 2020, and the first iteration of these was disseminated in July 2021 (ref. ^13^). To inform the guidelines update, a Guidelines Development Group for Screening and Treatment to Prevent Cervical Cancer was formed. The WHO consulted with methodologists and technical expert to determine the relevant research questions, timelines and methodology. Modeling was commissioned to support the work of the Guidelines Development Group and to quantify the benefits, harms and cost-effectiveness of potential screening strategies in the general population and in women living with HIV. Here we present the modeled assessment for the general population of women across 78 LMICs, and, in a companion paper, we present results for women living with HIV^14^.

## Results

We used the *Policy1-Cervix* platform, a well-established and extensively validated dynamic model of HPV transmission, vaccination, HPV type-specific natural history, cancer survival, screening, diagnosis and treatment^4,5,7,8,15–23^, to predict outcomes over the lifetime of females aged 10–84 years who turn 30 in 2030 (born in 2000) across all 78 LMICs (model schematically shown in Supplementary Fig. 1). We assessed the impact of seven screening algorithms, including primary VIA, primary cytology, primary HPV DNA with no triage or triage using HPV16/18 genotyping, colposcopy, cytology or VIA. In the base case, screening intervals of 3 years and 5 years were considered for primary VIA and cytology, and intervals of 5 years and 10 years were considered for primary HPV. Screening and triage test performance for the base case and the ranges assumed for sensitivity analysis were informed by updated systematic review evidence. In this normative analysis, we assumed that 70% of women attended each routine screen, and 90% of women complied with follow-up or treatment. We report on the cost and cost-effectiveness of each strategy as a cost per Healthy-Adjusted Life-Year (HALY) saved, assuming 0% discounting for effects and 3% discounting for costs^24^. Outcomes included reduction in cancer incidence and mortality, number of pre-cancer treatments needed to prevent a cervical cancer death (NNT), pre-term delivery events directly due to pre-cancer treatment and the incremental cost-effectiveness ratio (ICER, expressed as US$ per HALY saved). Table 1 summarizes our main findings and the policy impact of this research.

**Table 1:** Policy summary table

Background	Access to effective cervical cancer prevention in LMICs is currently limited, and the burden of cervical cancer is disproportionally experienced in LMICs. Only 9–11% of women in LMICs have ever had a screening test, and VIA is the primary screening test in most of these settings, which has low sensitivity in a programmatic setting. In 2020, the WHO launched a global strategy to eliminate cervical cancer as a public health problem and recommends ‘90–70–90’ intervention targets by 2030. These are that: (1) 90% of girls fully vaccinated against HPV by 15 years of age; (2) 70% of women screened using a high-performance test by 35 years of age and again by 45 years of age; and (3) 90% of women identified with cervical pre-cancer or invasive cervical cancer provided adequate treatment and care. To facilitate the implementation of the elimination strategy, the WHO updated its 2013 cervical screening and treatment guidelines in 2021. Guidance was provided by the Guidelines Development Group for Screening and Treatment to Prevent Cervical Cancer, which comprises a range of scientists, healthcare providers, implementers, ministry of health representatives, systematic reviewers, program implementation experts and representatives of civil society. The guidelines update was informed by a range of evidence sources, including an updated systematic review on screening test performance and treatment efficacy and a modeled evaluation using the *Policy1-Cervix* modeling platform.
Main findings and limitations	Primary HPV testing approaches, considering a range of screen-and-treat options or different triaging methods, were the most effective approaches to screening, reducing age-standardized cervical cancer mortality rates by 63% or more over a lifetime when offered at 5-yearly intervals and if 70% coverage is achieved. Primary HPV testing was also the most cost-effective screening approach. Strategies involving triaging HPV^+^ women before treatment (with 16/18 genotyping, cytology, VIA or colposcopy) had close-to-equivalent effectiveness to HPV screening without triage and resulted in fewer pre-cancer treatments. Screening with VIA or cytology every 3 years was less effective and less cost-effective than screening with HPV every 5 years. Furthermore, primary screening with VIA generated more than double the number of pre-cancer treatments compared to screening with HPV. As expected, offering HPV testing only twice in a lifetime was less effective than offering it every 5 years but still reduced age-standardized mortality rates by at least 41%.
Policy implications	Primary HPV testing is the most effective and cost-effective approach to cervical screening in LMICs. These findings have directly informed updated cervical screening and treatment guidelines by the WHO, published in 2021, which recommend using primary HPV screening in a screen-and-treat or screen-triage-and-treat approach, starting at the age of 30 years with screening every 5 years or 10 years for the general population of women. This primary screening approach allows for delivery models involving self-collected HPV samples and/or point-of-care testing, which should help to facilitate more effective screening and treatment solutions, co-designed with the community. However, the availability of affordable and appropriately clinically validated HPV and triage test technologies will be key to realizing the effective implementation of HPV screening, and, thus, realizing cervical cancer elimination, in LMICs.

### Cervical cancer incidence and mortality

**Fig. 1:**
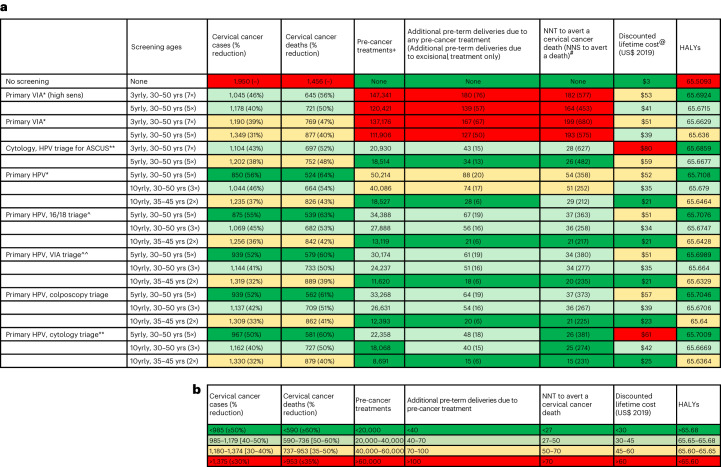
Predicted number of cervical cancer cases, cervical cancer deaths, pre-cancer treatments, additional pre-term delivery events, NNTs and NNSs, discounted costs and HALYs over the lifetime of a cohort of 100,000 women. **a**, Tabular summary of the lifetime number of cervical cancer cases, cervical cancer deaths, pre-cancer treatments, additional pre-term delivery events, number of pre-cancer treatments needed to prevent a cervical cancer death (NNT), costs and HALYs over the lifetime of a cohort of 100,000 women across 78 LMICs. The cells are colored to provide an overall impression of strategies that are performing well—best-performing strategies in a column are colored green (best = largest cancer incidence/mortality reduction or the lowest number of pre-cancer treatments, NNTs or costs)—followed by teal, yellow and then red for the worst-performing strategies. **b**, The range for the color-coding for each column. ASCUS, atypical squamous cells of undetermined significance; NNS, needed to screen; yrly, yearly; yrs, years. Outcomes represent total events over the lifetime of a cohort of 100,000 women. Percentage discounted costs represent costs incurred over the lifetime of an average woman discounting by 3% from age 30, as described in Methods. ‘]’ indicates that the percentage in the range is inclusive of the endpoints; ‘)’ indicates that it is not. *All positive women treated after assessment of eligibility for ablative treatment. **Triage positive referred to colposcopy. ^^VIA triage positive women treated after assessment of eligibility for ablative treatment. ^HPV 16/18 positive women treated after assessment of eligibility for ablative treatment. Women positive for HPV types other than HPV 16/18 (‘OHR’) are triaged with VIA. +There could be multiple treatments in the same woman over her lifetime. ^@^0% discount rate for effect, 3% discount rate for cost.

In the absence of further intervention (no screening), over the lifetime of a cohort of 100,000 unscreened women in 78 LMICs, 1,950 cervical cancer cases and 1,456 deaths are predicted to occur (Fig. 1), and the average age-standardized cervical cancer incidence rate (ASIR) and mortality rate (ASMR) would be 19.8 and 14.1 per 100,000 women, respectively (not shown).

**Fig. 2:**
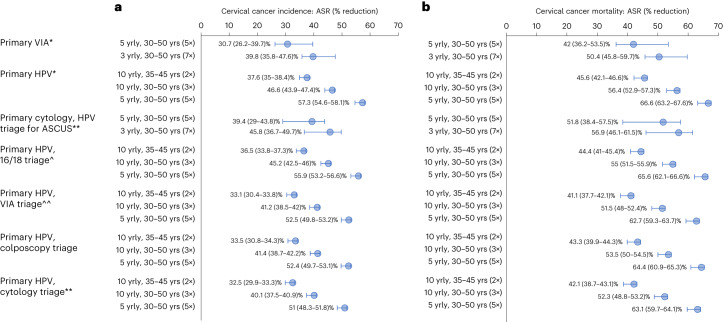
Impact of screening scenarios on cervical cancer incidence and mortality rates. Reductions in age-standardized cervical cancer incidence (**a**) and age-standardized cervical cancer mortality (**b**) compared to no screening, shown as the dots for base case assumptions. The error bars represent the reductions when assuming the best (upper range) and worst (lower range) primary test performance assumptions, as described in Supplementary Table 3. Age-standardization was performed using the 2015 World Female Population for ages 0–99 years. ASCUS, atypical squamous cells of undetermined significance; yrly, yearly; yrs, years. *All positive women treated after assessment of eligibility for ablative treatment. **Triage positive referred to colposcopy. ^^VIA triage positive women treated after assessment of eligibility for ablative treatment. ^HPV 16/18 positive women treated after assessment of eligibility for ablative treatment. Women positive for HPV types other than HPV 16/18 (‘OHR’) are triaged with VIA. ^o^The range in sensitivity to CIN2+ is varied as shown in Supplementary Table 3c: for primary HPV, we consider a range of CIN2+ sensitivity of 88% (worst case) to 96% (best case) for primary cytology, we consider a range of CIN2+ sensitivity at the LSIL threshold of 46.8% (worst case) to 80% (best case) and for primary VIA, we consider a range of CIN2+ sensitivity of 30% (worst case) to 60% (best case).

In the base case analysis, primary HPV testing without triage every 5 years for ages 30–50 years was the most effective strategy, with a 57% reduction in ASIR and a 67% reduction in ASMR compared to no screening (rounded to nearest whole percentage point; Figs. 1 and 2). Strategies involving triage of HPV^+^ women before treatment reduced ASIR by 51–56% and ASMR by 63–66% (range dependent on triage test). Primary cytology every 3 years could reduce ASIR by 46% and ASMR by 57%. In the base case, when incorporating best-fitted assumptions for VIA sensitivity of 41% for CIN2+ (Methods), primary VIA testing every 3 years could reduce ASIR by 40% and ASMR by 50%. Even if VIA could achieve sustained, population-level sensitivity to CIN2+ of 60% (which was evaluated in sensitivity analysis as a favorable assumption), primary VIA testing every 3 years for ages 30–50 years would reduce ASIR by 48% and ASMR by 60% and, thus, remains less effective than primary HPV testing every 5 years. Primary HPV screening every 10 years for ages 35–45 years (twice-lifetime screening) with or without triage—the scenario considered for the CCEMC elimination modeling—could reduce ASIR by at least 33% and ASMR by at least 41%, representing two-thirds of the benefits seen with 5-yearly HPV testing.

**Extended Data Fig. 1:**
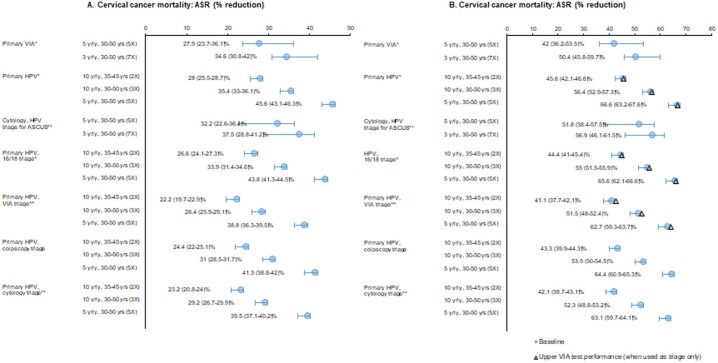
Cervical cancer mortality ASR % reductions (A) assuming lower screening adherence^o^ and (B) assuming favorable VIA triage test performance (black triangles). The dots represent reductions assuming base case assumptions for test performance and the error bars represent the reductions when assuming the best (upper range) and worst (lower range) primary test performance assumptions as described in Supplementary Table 3. ^o^Assuming 50% attendance for routine screening (with 30% of women never attending) and 75% for adherence with treatment or follow-up visits (100% for same-day eligibility). *All positive women treated after assessment of eligibility for ablative treatment. **Triage positive referred to colposcopy. ^^VIA triage positive women treated after assessment of eligibility for ablative treatment. ^HPV 16/18 positive women treated after assessment of eligibility for ablative treatment. Women positive for HPV types other than HPV 16/18 (‘OHR’) are triaged with VIA.

**Extended Data Fig. 2:**
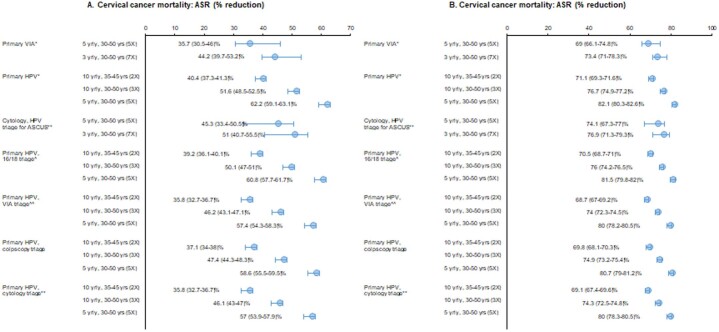
Cervical cancer mortality ASR % reductions (A) assuming both women symptomatically-detected and screen-detected for cancer receive status-quo rates for cancer treatment access and (B) assuming both women symptomatically-detected and screen-detected for cancer receive 90% cancer treatment access. The dots represent reductions assuming base case assumptions for test performance and the error bars represent the reductions when assuming the best (upper range) and worst (lower range) primary test performance assumptions as described in Supplementary Table 3. *All positive women treated after assessment of eligibility for ablative treatment. **Triage positive referred to colposcopy. ^^VIA triage positive women treated after assessment of eligibility for ablative treatment. ^HPV16/18 positive women treated after assessment of eligibility for ablative treatment; women positive for HPV types other than HPV 16/18 (‘OHR’) are triaged with VIA.

If only 50% of women attend primary screening and 75% comply with follow-up, primary HPV testing remained the most effective screening approach, although absolute reductions in cervical cancer incidence and mortality rates were correspondingly lower across all scenarios (Extended Data Fig. 1). Primary HPV screening also remained the most effective approach under different assumptions about primary and triage test performance (Extended Data Fig. 1) and cancer treatment access (Extended Data Fig. 2).

### Balance of benefits and harms

Over the lifetime of a cohort of 100,000 women, primary HPV screening without triage every 5 years for ages 30–50 years was predicted to result in 50,214 pre-cancer treatments and 88 additional pre-term delivery events, and the NNT to prevent a cervical cancer death was predicted to be 54 (Fig. 1). Triaging HPV^+^ women before treatment with either VIA, HPV16/18 genotyping, cytology or colposcopy would generate 32–55% fewer pre-cancer treatments and generated 26–37 NNTs to prevent a cervical cancer death.

**Fig. 3:**
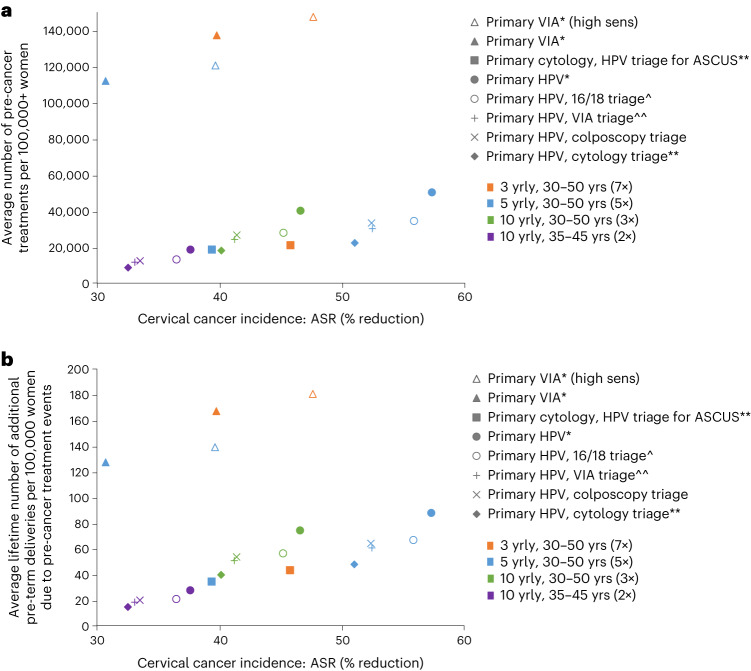
Comparison of age-standardized cervical cancer incidence reduction as a measure of the benefits-to-harms profile of each strategy. **a**, Lifetime number of pre-cancer treatments. **b**, Lifetime number of additional pre-term deliveries due to pre-cancer treatment. ASCUS, atypical squamous cells of undetermined significance; yrly, yearly; yrs, years. +There could be multiple treatments in the same woman over her lifetime. *All positive women treated after assessment of eligibility for ablative treatment. **Triage positive referred to colposcopy. ^^VIA triage positive women treated after assessment of eligibility for ablative treatment. ^HPV 16/18 positive women treated after assessment of eligibility for ablative treatment. Women positive for HPV types other than HPV 16/18 (‘OHR’) are triaged with VIA.

When assuming base case assumptions for VIA test performance, primary VIA screening resulted in the highest number of pre-cancer treatments overall, with more than 110,000 pre-cancer treatments predicted over the lifetime of the cohort of 100,000 women—more than double the lifetime number of pre-cancer treatments compared to any of the primary HPV or primary cytology strategies (Figs. 1 and 3). Primary VIA screening also generated at least 127 additional pre-term deliveries over the lifetime of the cohort. The NNT to prevent a cervical cancer death was more than 190 for primary VIA strategies, which is nearly four times more than that predicted for any of the primary HPV testing strategies.

In sensitivity analysis, we explored the assumption that only excisional treatment can cause additional pre-term delivery events (that is, that ablation does not result in additional pre-term deliveries). With this assumption, the number of additional pre-term deliveries is predicted to be 58–79% lower than equivalent scenarios under base case assumptions (Fig. 1).

### Cost-effectiveness

**Fig. 4:**
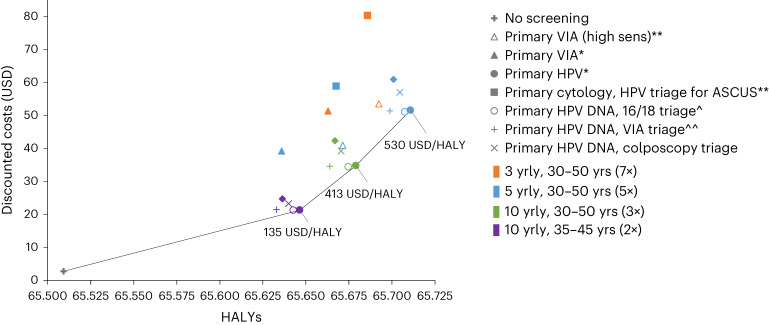
Cost-effectiveness plane depicting relationship between cost and HALYs for each screening strategy. The results are shown for alternative primary screening and triaging options and for different relevant screening intervals and age ranges. For those strategies appearing on the cost-effectiveness frontier, the incremental cost-effectiveness ratio is noted (cost per HALY). ASCUS, atypical squamous cells of undetermined significance; USD, US dollar ($); yrly, yearly; yrs, years. *All positive women treated after assessment of eligibility for ablative treatment. **Triage positive referred to colposcopy. ^^VIA triage positive women treated after assessment of eligibility for ablative treatment. ^HPV 16/18 positive women treated after assessment of eligibility for ablative treatment. Women positive for HPV types other than HPV 16/18 (‘OHR’) are triaged with VIA. +0% discount rate for effect, 3% discount rate for cost. As a reference point for a potential WTP threshold across 78 LMICs, the population-weighted average GDP per capita (pc) for 2019 across the 78 LMIC is US$2,093, and 69 of 78 (89%) of LMICs had a GDP pc equal to or above US$530 and 77/78 (99%) of LMICs had a GDP pc equal to or above US$136.

**Extended Data Fig. 3:**
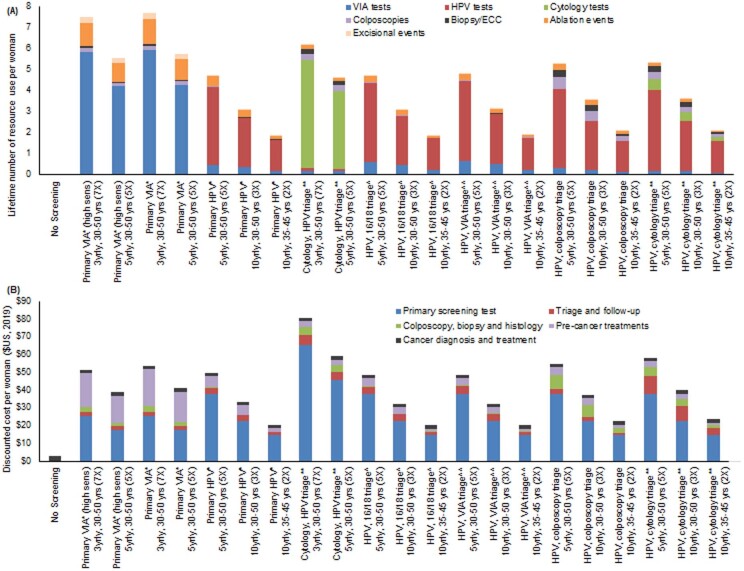
(A) Resource utilization breakdown for each strategy and (B) average discounted cost per woman in the population, over the lifetime of the cohort, broken down by component for each strategy. @3% discount rate for cost. *All positive women treated after assessment of eligibility for ablative treatment. **Triage positive referred to colposcopy. ^^VIA triage positive women treated after assessment of eligibility for ablative treatment. ^HPV16/18 positive women treated after assessment of eligibility for ablative treatment; women positive for HPV types other than HPV 16/18 (‘OHR’) are triaged with VIA.

In the base case analysis, primary HPV screening without triage was on the cost-effectiveness frontier and had an ICER of US$530 per HALY saved when screening every 5 years for ages 30–50 years, an ICER of US$413 per HALY saved when screening every 10 years for ages 30–50 years and an ICER of US$135 per HALY saved when screening every 10 years for ages 35–45 years (twice-lifetime screening) (Fig. 4). Primary HPV screening using any triaging strategy was generally near the cost-effectiveness frontier. Under base case assumptions, primary VIA and primary cytology screening strategies were furthest from the cost-effectiveness frontier and were the least cost-effective strategies. For primary HPV strategies, more than 60% of the (discounted) costs experienced over the lifetime of the cohort are from the primary HPV test cost alone (Extended Data Fig. 3). As a reference point for a potential willingness-to-pay (WTP) threshold across 78 LMICs, 69 of 78 (89%) LMICs had a gross domestic product (GDP) per capita equal to or above that predicted for 5-yearly HPV testing, the strategy along the frontier with the highest ICER (US$530 per HALY), and 77 of 78 (99%) of LMICs had a GDP per capita equal to or above that predicted for primary HPV screening every 10 years for ages 35–45 years (twice-lifetime screening).

**Fig. 5:**
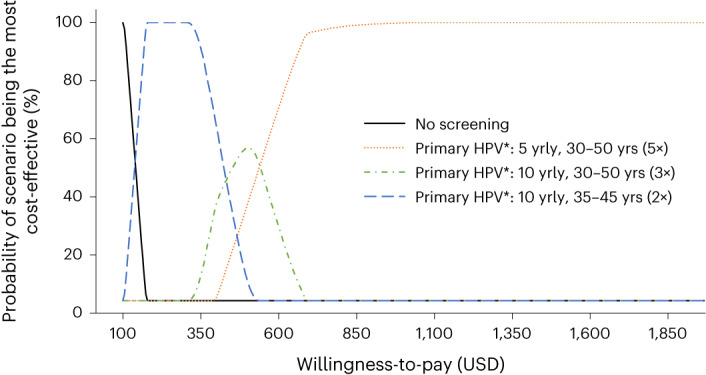
Cost-effectiveness acceptability curves. These curves show the probability of a strategy being the most cost-effective for a range of WTP values of US$100–$2,000 per HALY. USD, US dollar ($); yrly, yearly; yrs, years. *All positive women treated after assessment of eligibility for ablative treatment. Some strategies had a small probability of being cost-effective but are not visible on the graph and are as follows: primary VIA screening (high sensitivity) every 5 years for ages 30–50 had <4% probability of being the most cost-effective approach for WTP US$290–$570/HALY saved. Primary VIA screening (high sensitivity) every 3 years for ages 30–50 had <5% chance of being the most cost-effective approach for WTP US$480–$1,095/HALY saved. Primary HPV with HPV16/18 triage every 5 years for ages 30–50 had <0.1% chance of being the most cost-effective approach for WTP US$410–$440/HALY saved.

**Extended Data Fig. 4:**
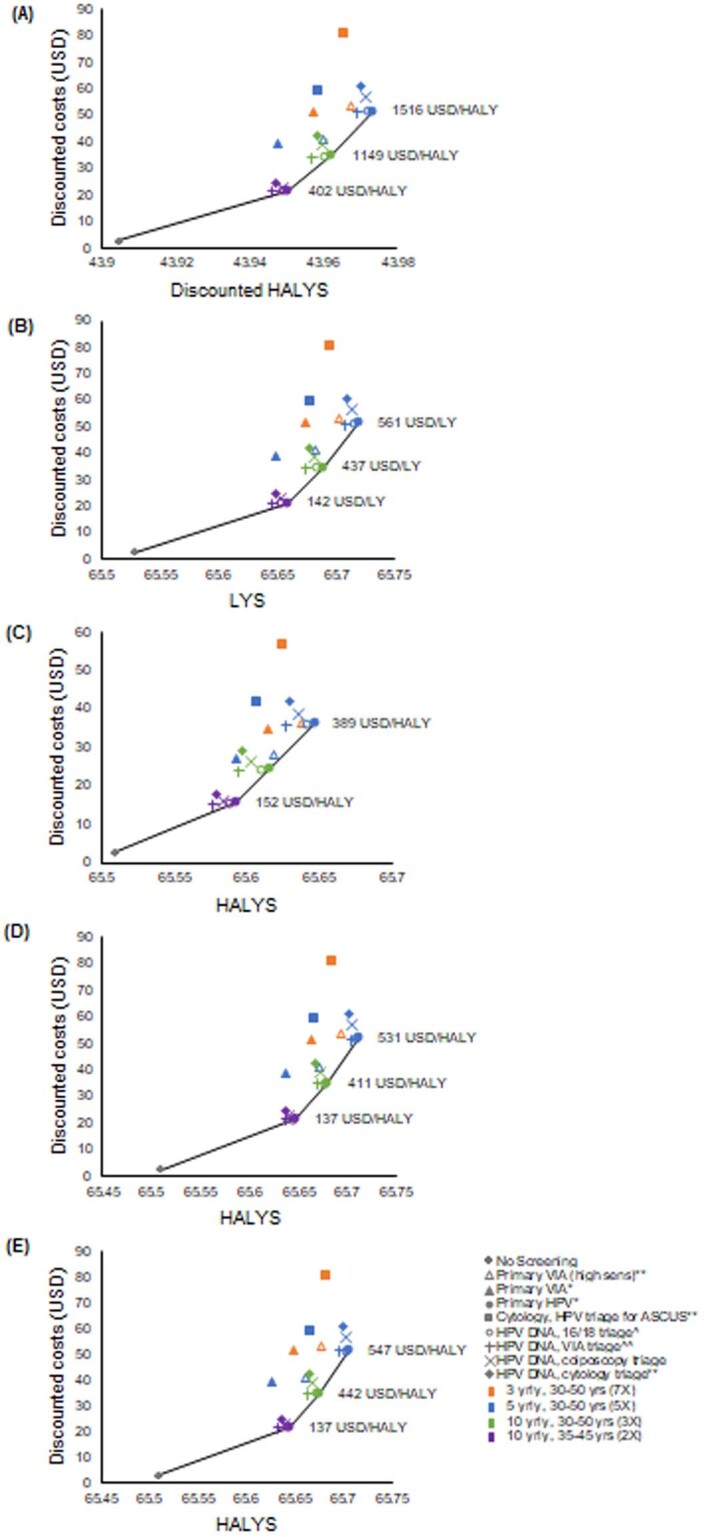
Cost-effectiveness outcomes assuming (A) 3% discount for both costs and effects; (B) using life-years without disability weights; (C) assuming low screening adherence; and (D) assuming favorable VIA performance; (E) including pre-cancer treatment disutilities for HALYs calculation. *All positive women treated after assessment of eligibility for ablative treatment. **Triage positive referred to colposcopy. ^^VIA triage positive women treated after assessment of eligibility for ablative treatment. ^HPV16/18 positive women treated after assessment of eligibility for ablative treatment; women positive for HPV types other than HPV 16/18 (‘OHR’) are triaged with VIA.

**Extended Data Fig. 5:**
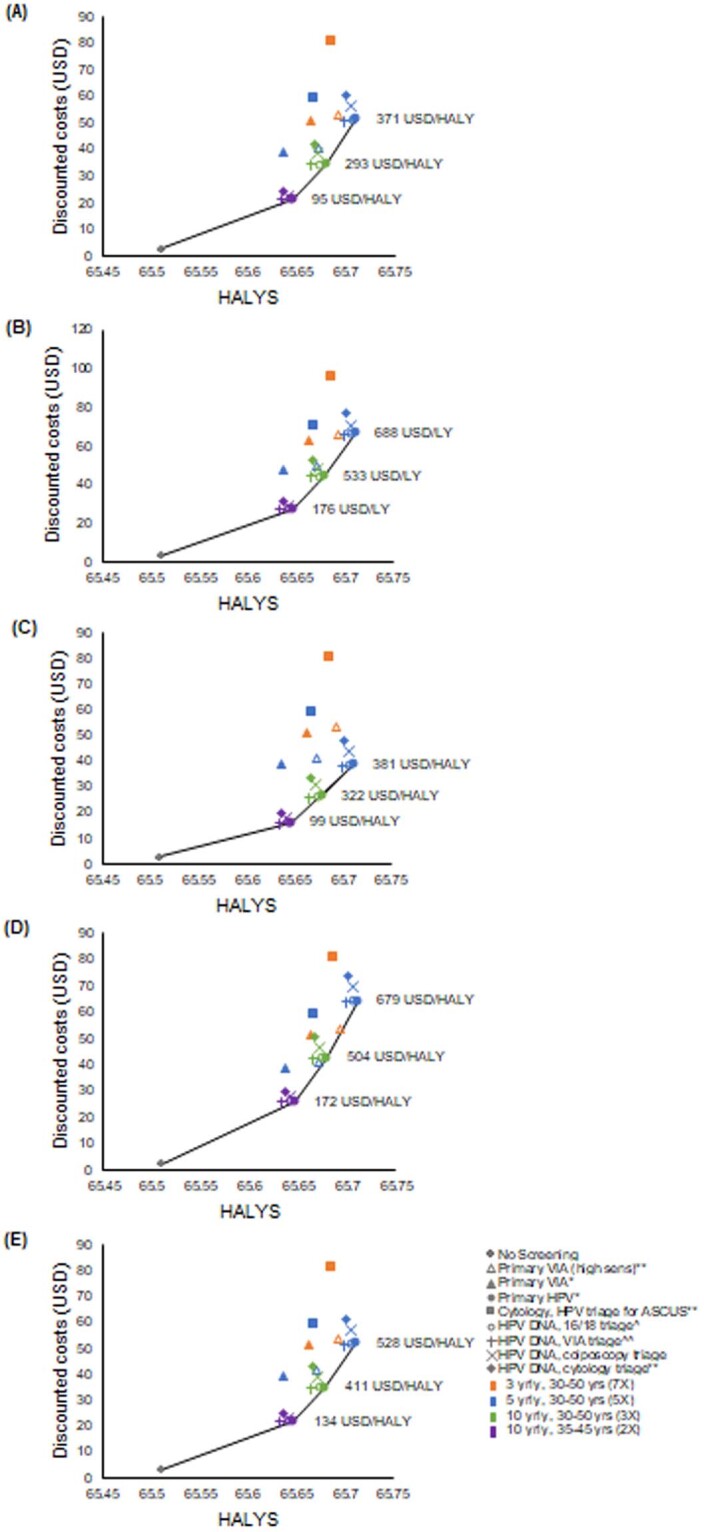
Cost-effectiveness across 78-LMICs assuming (A) Lower bound assumptions for all costs (B) Upper bound assumptions for all costs (C) Lower bound assumptions for HPV test costs (all other costs at base case) (D) Upper bound assumptions for HPV test costs (all other costs at base case) (E) Upper bound assumptions for Cancer treatment costs (all other costs at base case). *All positive women treated after assessment of eligibility for ablative treatment. **Triage positive referred to colposcopy. ^^VIA triage positive women treated after assessment of eligibility for ablative treatment. ^HPV16/18 positive women treated after assessment of eligibility for ablative treatment; women positive for HPV types other than HPV 16/18 (‘OHR’) are triaged with VIA.0% discount rate for effect, 3% discount rate for cost HALY: health-adjusted life-years.

When considering varying discount rates, disability weights, test performance, screening coverage assumptions and cost values in sensitivity analysis, we found that primary HPV screening remained the most cost-effective strategy, but the ICERs varied from the base case (Extended Data Figs. 4 and 5). Acceptability curves depicting the percentage of the probabilistic sensitivity analysis (PSA) samples that yielded each scenario as most cost-effective as a function of the WTP threshold are shown in Fig. 5. For WTP under US$140, the status quo (that is, no screening) was found to have the highest probability of being the most cost-effective strategy. For WTP between US$140 and US$420, primary HPV screening every 10 years for ages 35–45 years (twice-lifetime screening) had the highest probability of being the most cost-effective; for WTP between US$425 and US$520, primary HPV screening every 10 years from ages 30–50 years had the highest probability of being the most cost-effective; and for WTP more than US$525, primary HPV screening every 5 years from ages 30–50 years had the highest probability of being the most cost-effective. As noted earlier, the most cost-effective HPV strategy involved no triage; however, strategies involving primary HPV with triaging were close to the cost-effectiveness frontier.

**Extended Data Fig. 6:**
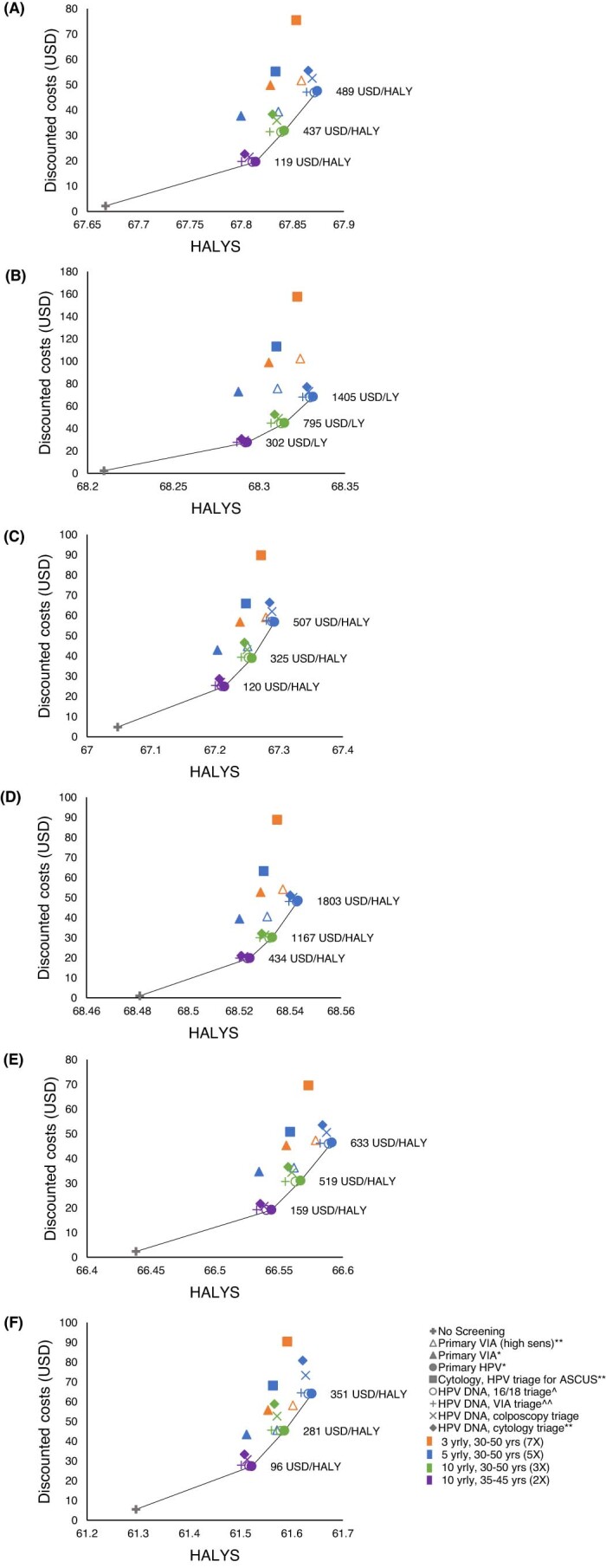
Cost-effectiveness for different regions (A) East Asia & Pacific; (B) Europe & East Asia; (C) Latin America & Caribbean; (D) Middle East & North Africa; (E) South Asia; (F) Sub-Saharan Africa. *All positive women treated after assessment of eligibility for ablative treatment. **Triage positive referred to colposcopy. ^^VIA triage positive women treated after assessment of eligibility for ablative treatment. ^HPV16/18 positive women treated after assessment of eligibility for ablative treatment; Women positive for HPV types other than HPV 16/18 (‘OHR’) are triaged with VIA.0% discount rate for effect, 3% discount rate for cost. HALY: health-adjusted life-years.

When considering regional-level results in exploratory analysis, primary HPV screening without triage remained on the cost-effectiveness frontier for each region, and triaging HPV^+^ women before treatment remained close to the frontier, and sometimes appeared on the frontier for some regions (Extended Data Fig. 6). For 5-yearly primary HPV screening without triage, Europe & Central Asia, Middle East & North Africa and South Asia had higher ICERs ($US1405 per HALY, $US1803 per HALY and $US633 per HALY, respectively) than the average 78 LMIC value. Conversely, East Asia & Pacific, Latin America & Caribbean and Sub-Saharan Africa had lower ICERs ($US489 per HALY, $US507 per HALY and $US351 per HALY, respectively) than the average 78 LMIC value. The differences were largely driven by accounting for higher HALYs in high-burden-of-disease countries, making screening more cost-effective in these settings. Overall, this exploratory analysis of regional-level outputs produced similar results to those for the all 78 LMICs aggregate.

### Additional analyses—management of triage-negative women

**Extended Data Fig. 7:**
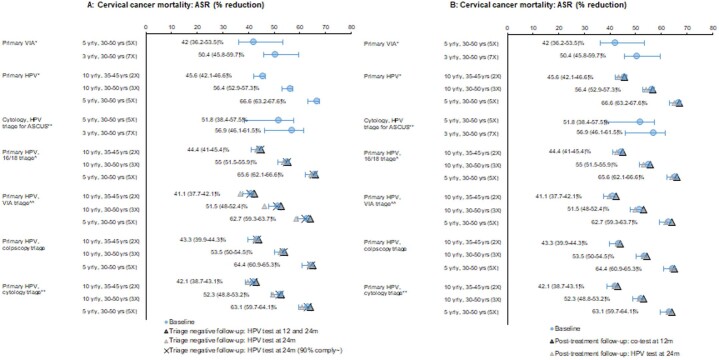
Cervical cancer mortality ASR % reductions assuming (A) HPV positive and triage negative women are followed-up after 2 years with 10% loss-to-follow-up (black triangle), followed-up after 2 years with 30% loss-to-follow-up (grey triangle), followed-up at both 1- and 2- years with 10% loss-to-follow-up for each visit (black crosses)) and (B) assuming women treated for pre-cancer treatment (not known to have CIN3 + ) are followed-up after 2 years assuming with 30% loss-to-follow-up (gray triangle), followed-up after 1 year with cotesting assuming 10% loss-to-follow-up (black triangle). The dots represent reductions assuming base case assumptions for test performance and the error bars represent the reductions when assuming the best (upper range) and worst (lower range) primary test performance assumptions as described in Supplementary Table 3. *All positive women treated after assessment of eligibility for ablative treatment. **Triage positive referred to colposcopy. ^^VIA triage positive women treated after assessment of eligibility for ablative treatment. ^HPV16/18 positive women treated after assessment of eligibility for ablative treatment; women positive for HPV types other than HPV 16/18 (‘OHR’) are triaged with VIA.

**Extended Data Fig. 8:**
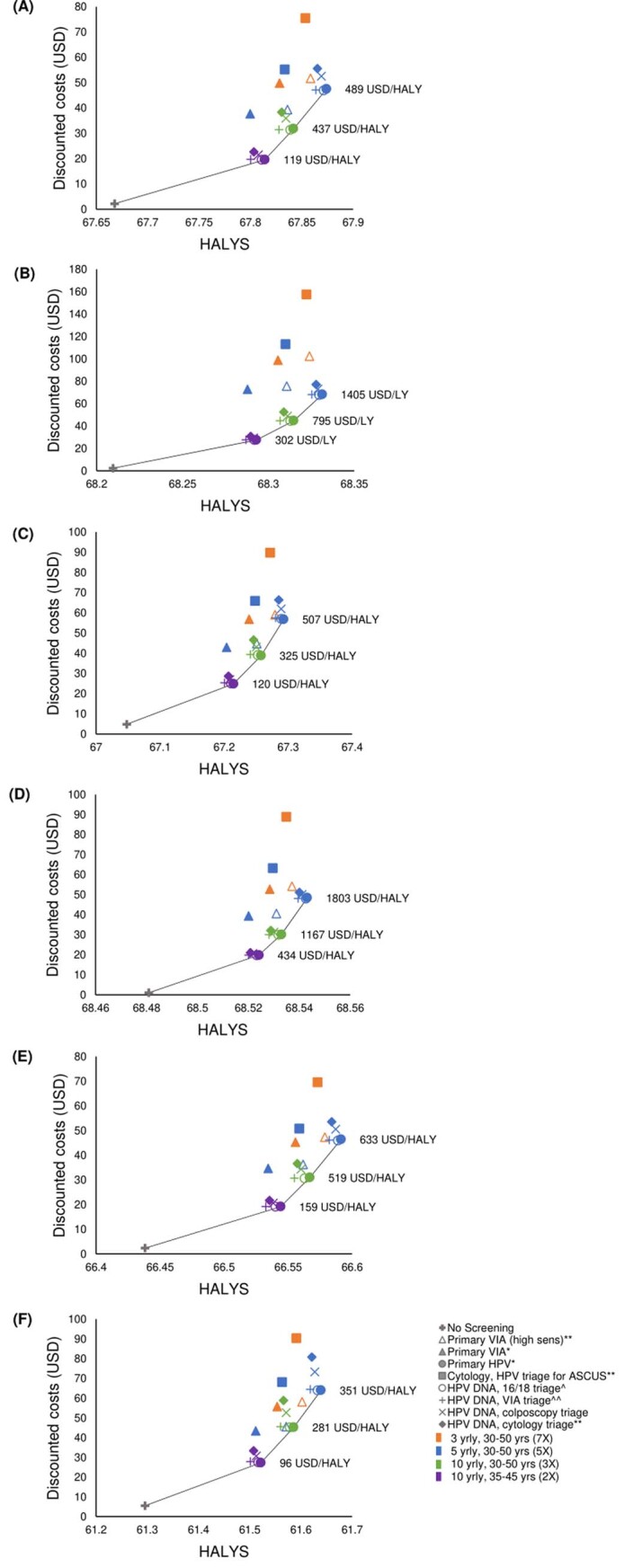
Lifetime number of precancer treatment events for each strategy, including different management of HPV positive women who have a negative triage, versus (A) Cervical cancer incidence ASR % reduction and (B) Cervical cancer mortality ASR % reduction. *All positive women treated after assessment of eligibility for ablative treatment. **Triage positive referred to colposcopy. ^^VIA triage positive women treated after assessment of eligibility for ablative treatment. ^HPV16/18 positive women treated after assessment of eligibility for ablative treatment; women positive for HPV types other than HPV 16/18 (‘OHR’) are triaged with VIA. +Note there could be multiple treatments in women who require follow-up.

In the base case, for strategies involving primary HPV testing with triage before treatment, we assumed that women who tested HPV-positive and triage-negative would return in 12 months for an HPV test, and would then return to routine screening (or be discharged if outside of the screening age range) if negative at this visit. When assuming that women who are primary HPV test positive and triage negative are followed-up in 24 months instead of 12 months but that loss-to-follow-up remained at 10% (less aggressive management; Extended Data Fig. 7), there was less than 1% difference in ASMR versus the base case (ranges represent variations across different screening frequencies and triage strategies). We also found that this management resulted in a 5–22% decrease in pre-cancer treatments and a 5–23% reduction in NNTs to prevent a cervical cancer death (Extended Data Fig. 8). However, if we assumed a 30% loss-to-follow-up at the 24-month visit, there was a 1–9% increase in ASMR versus base case, with the greatest increase observed when VIA was used as the triage test. When assuming women who are primary HPV test positive and triage negative are followed-up at 12 months and 24 months and require a negative HPV test at both visits before being discharged from follow-up (more aggressive management), there was a 1–4% decrease in ASMR versus the base case assumption of one visit at 12 months only (Extended Data Fig. 7).

### Additional analyses—management of women treated for pre-cancer

In the base case, we assumed that women treated for cervical pre-cancer who did not have a histological diagnosis of CIN3 would return in 12 months for an HPV test and be returned to routine screening (or discharged if outside of the screening age range) if negative at this visit. When assuming women who are treated for pre-cancer that is not known to be CIN3 are followed-up with a single visit at 24 months with 30% loss-to-follow-up instead of the base case assumption of 12 months (less aggressive management; Extended Data Fig. 7), at a population level, there was a 1–2% increase in ASMR. When assuming women who are treated for pre-cancer that is not known to be CIN3 are followed-up with a single visit at 12 months with co-testing instead of HPV testing alone (more aggressive management), there was a 1–2% decrease in ASMR (Extended Data Fig. 7).

## Discussion

We performed a modeled assessment of the benefits, harms and cost-effectiveness of seven primary screening scenarios across 78 LMICs for the general population of women over their lifetime. We found that primary HPV screening would result in the greatest reductions in cervical cancer incidence and mortality, would optimize the balance of benefits to harms and would be cost-effective compared to other primary testing approaches. The findings presented here have directly informed updated cervical screening and treatment guidelines by the WHO, published in 2021 (ref. ^13^), which recommend primary HPV screening in a screen-and-treat or screen-triage-and-treat approach, starting at age 30 years with screening every 5 years or 10 years for the general population of women. Modeling for women living with HIV was performed separately and is presented in a companion manuscript^14^.

We found that settings that can support 5-yearly HPV screening and achieve coverage rates of 70% for ages 30–50 years would experience a 50% or greater reduction in cervical cancer incidence and a 60% or greater reduction in cervical cancer mortality. Primary HPV testing every 5 years was more effective than primary VIA or cytology every 3 years, and, compared to VIA, primary HPV testing generated substantially fewer pre-cancer treatments, even when favorable assumptions were made around the performance of VIA testing. We found that primary HPV testing could result in up to 88 additional pre-term delivery events over the lifetime of 100,000 women but that the number of additional pre-term delivery events would more than double with VIA screening. We found that primary HPV testing without triage was the most effective approach; however, if high rates of follow-up can be achieved, triaging HPV^+^ women before treatment had close-to-equivalent effectiveness and had the capacity to reduce pre-cancer treatment rates and additional pre-term delivery events. There will likely be high rates of detection of prevalent pre-cancer and invasive cancer in the first round of screening with HPV (because it is a highly sensitive test). However, in the second and subsequent rounds of screening, the detected disease will be more likely to be incident, and, thus, the balance of benefits to harms may change and may be less favorable to HPV screen without triaging. Emerging technologies, such as Automated Visual Evaluation, when used as a triage, could play a role in reducing the harms associated with pre-cancer treatment of HPV^+^ women in subsequent rounds and will be an important consideration in future^25^.

We found that triaging with low-sensitivity VIA was effective in the context of primary HPV screening. This is because any underlying pre-cancer or cancer in HPV^+^ women, which is missed by VIA triage, has potential to be detected by HPV testing at the 12-month follow-up; thus, the high effectiveness of this screening scenario is highly dependent on the assumption that women who are negative on a triage test have 90% compliance with a repeat HPV test in 12 months, followed by immediate ablation for eligible women if persistently HPV positive. In additional analyses, we found that, if only 70% of women return after testing triage negative, the effectiveness of the program could be substantially reduced, particularly if VIA was used as the triage test. It will, thus, be critical to establish screen-triage-and-treat programs in the context of an investment in integrated data systems for maintaining high follow-up rates for women referred for surveillance. It has previously been found, for example, that establishing a digital registry system to support an HPV screening program in Malaysia, at a cost of US$8.50 per woman, would be effective and cost-effective if it increased adherence with follow-up from lower rates of 50% to 75% to higher rates of 90%^26^. In general, our findings support the generalized use of primary HPV screening, but the choice of triaging strategy, the start age (30 years or 35 years) and the screening interval (whether 5 years or 10 years) need to be contextualized to the country or setting and will depend, in part, on resourcing and local considerations for the benefits-versus-harms profile for screening.

Cervical cancer elimination modeling performed by the CCEMC, which included results from *Policy1-Cervix*, found that, if the 2030 triple-intervention targets are achieved in 78 LMICs, cervical cancer would be eliminated in all LMICs, and a total of 74.1 million cancer cases and 62.6 million deaths would be averted over the course of the century^4,5^. The framing of these earlier analyses is different to what is presented here. These earlier analyses focused on the impact of three sets of interventions—vaccination, screening and cancer treatment—and assumed that screening would be offered twice in a lifetime. However, these earlier evaluations did not focus on the detailed options available for cervical screening, including alternative management pathways, triage test options and numbers of screening tests that a woman could be offered over a lifetime, which is the subject of the current evaluation. In the current analysis, we found that primary HPV screening without triage at 35 years and again at 45 years—equivalent management to the earlier elimination analysis—could reduce cervical cancer cases by 37% and deaths by 43% over the lifetime of a cohort of women, which is about two-thirds of the reductions predicted by 5-yearly screening for ages 30–50 years. The implication is that elimination timing will be sooner, and total cases and deaths averted will be greater, if primary HPV screening is implemented at 5-yearly intervals for ages 30–50 years as opposed to 10-yearly intervals at ages 35 years and 45 years (that is, twice-lifetime).

A strength of the *Policy1-Cervix* platform is that it captures a high level of detail in screening management algorithms, including downstream management of HPV^+^ women and surveillance after colposcopy and pre-cancer treatment. We previously used this capacity of *Policy1-Cervix* to inform the transition from cytology to primary HPV screening within the National Cervical Screening Program in Australia^7^ as well as similar screening policy evaluations for New Zealand^8^ and England^17^. Another strength is that, throughout the evaluation, the modeling team regularly met with members of the Guidelines Development Group and relevant technical teams to agree on key parameters and assumptions and discuss the interpretation of results. We considered screening and triage technologies for which there was a sufficient evidence base to support modeling, and we relied on updated systematic reviews that built upon a recent major review of the evidence^12^. The WHO guidelines were updated in 2021 to include guidance on use of primary HPV mRNA testing, for which further modeling analyses were performed using *Policy1-Cervix*^27,28^. Alternative or emerging triage approaches, including dual-stain cytology, are also being considered in subsequent iterations for the living guidelines.

This analysis has some limitations. We assessed the outcomes of different screening approaches for all women living in LMICs regardless of HIV status. For many countries, the proportion of women living with HIV is very small; however, some LMICs have high HIV prevalence. For instance, more than one-quarter of women ages 15–49 years in Lesotho are living with HIV^29^. Therefore, a companion analysis that explicitly captures the interaction with HIV and HPV has evaluated cervical screening in women living with HIV^14^. Another limitation of the analysis is that data on the relative risk of adverse obstetric outcomes (used to inform calculations of additional pre-term delivery events) are derived predominantly from high-income countries, and there are considerable uncertainties in the application to LMICs and potential differences for ablation versus excision and by depth of excision^30^. Our analysis at this stage could not consider costs incurred by wider society, including productivity losses and out-of-pocket costs. Also, the cost of assays and clinical procedures and care are highly variable between countries, and future market forces and other factors could have a substantial impact on cost estimates. However, to capture overall uncertainties in costs, we considered uncertainty in almost all costing components in one-way sensitivity analysis and probabilistic sensitivity analysis and found that primary HPV testing remained the most cost-effective approach. Our natural history model has been validated against a range of data sources; however, there are still some uncertainties in unobservable natural history parameters. We previously found that primary HPV screening remains the most cost-effective approach when considering uncertainties in natural history parameters in high-income settings^7^.

We assumed that screening would be offered to women aged 30–50 years, which was directly informed through discussions with the Guidelines Development Group. One consideration for the upper age range was the higher rate of comorbidities and lower life expectancy in women aged over 50 years in LMICs, thereby reducing the amount of disability-free life-years that could be gained by screening older women. Additionally, inadequate visualization of the transformation zone is typical after menopause, and ablative treatment is not suitable for treatment in situations in which the transformation zone is not visible. The WHO approach to continuous re-evaluation of the guidelines (‘living guidelines’) means that screening end-age could potentially be reconsidered in future. It should also be noted that, by design, the current analysis did not explicitly consider the impact of screening in women who had been offered HPV vaccination as adolescents. Some LMICs have implemented HPV vaccination, and some have already achieved high coverage. Twelve LMICs have introduced a national HPV vaccination program (most of these started after 2017)^31^. However, even if high coverage could be reached and maintained in these countries and rapidly scaled-up for 9–14-year-olds across all other 78 LMICs, it will be at least 10–20 years before these females reach an age eligible for screening, and it will be 30–40 years before all screen age eligible women (ages 30–50 years) are likely to have been offered HPV vaccination as adolescents (even in the best case). Therefore, for the next few decades, cervical screening recommendations for unvaccinated women will be most relevant to LMICs. We previously assessed optimal screening management for cohorts offered HPV vaccination in high-income countries, which commenced HPV vaccination up to 15 or more years previously, in which vaccinated cohorts have already entered screening programs, and we found that primary HPV testing remained the optimal approach but that the number of screens required in a lifetime could be reduced^18^.

Understanding social and cultural barriers is crucial to achieving high coverage for cervical screening. There are substantial resourcing and financing challenges associated with scaling-up HPV testing in LMICs, including supply and delivery challenges for validated screening tests^32^ and health system and infrastructure challenges associated with setting up referral pathways for diagnosis and treatment for more advanced lesions. However, the coronavirus disease 2019 (COVID-19) pandemic has led to widespread dissemination of testing platforms compatible with HPV testing, which could help facilitate scale-up of HPV screening. Integrating screening programs with existing primary care services—for example, by offering HPV testing at sexual health clinics, ante-natal care consultations or family planning consultations—will also facilitate access to screening. Integration of HPV testing into existing community outreach centers for HIV control has been shown, for example, to result in high screening uptake in Zimbabwe^33^.

Our results for primary HPV screening can be taken to apply to a wide range of clinically validated HPV tests, including technologies allowing point-of-care (PoC) testing and also self-collected testing, which has been shown to approach test performance observed for clinician-collected samples if polymerase chain reaction (PCR)-based testing is used^34^. Offering PoC HPV testing in certain settings could reduce loss to follow-up if pre-cancer treatment can be performed in the same visit. Offering self-collection has the potential to greatly increase the acceptability of screening and may help achieve high coverage^35^. Successful experiences have been documented with the use of primary HPV testing with PoC self-collection and same-day ablative treatment in LMICs; for example, this has been found to be acceptable, effective and cost-effective in Papua New Guinea as part of a program to eliminate cervical cancer in the Western Pacific^36–38^.

Overall, our findings support the updated cervical WHO screening and treatment guidelines. The guidelines are a critical enabler of the global strategy to accelerate the elimination of cervical cancer as a public health problem. To further support scaling-up cervical screening in LMICs, the WHO has also released updated guidelines for pre-cancer treatment^39^. Cervical screening and treatment have also been identified by the WHO as ‘best buys’ in cancer control for Member States^40^. The elimination strategy is a component of the United Nations Global Strategy for Women’s, Children’s and Adolescent’s Health, and investment in cervical cancer elimination will support several sustainable development goals (SDGs) and targets, including SDG 3 (good health and well-being), SDG 5 (gender equity) and SDG 10 (reducing inequalities). Ultimately, the WHO elimination strategy has been shown to be cost-effective^3^ and will prevent over 62 million deaths in LMICs over the next century^5^.

## Methods

### Evaluation process

The WHO’s updated cervical screening guidelines were informed by a range of evidence sources, including an updated systematic review on screening test performance and treatment efficacy and a modeled evaluation. To guide this update, the WHO secretariat formed the Guidelines Development Group and consulted with methodologists and technical groups to determine Problem/Population, Intervention, Comparison, Outcome (PICO) questions, timelines and methodology.

The updated guideline addressed optimal primary and triage test technologies, optimal screening frequency and intervals, optimal management of women under surveillance after testing triage negative at the primary visit and optimal management of women after treatment for pre-cancer. The modeling evaluation for both the general population of women and women living with HIV assesses a range of primary and triage test technologies and a range of screening ages and frequencies, incorporating data on updated systematic review evidence, costing data and screening algorithms.

The Guidelines Development Group identified seven priority algorithms that would be potentially suitable for LMICs: primary VIA, primary cytology with HPV triage (ASC-US referral), primary HPV without triage (all positive women treated after using assessment of eligibility for ablative treatment), primary HPV 16/18 triage, primary HPV VIA triage, primary HPV cytology triage and primary HPV colposcopy triage screening strategies (Supplementary Table 1). To ensure adequate communication among the different expert groups involved in informing the update of cervical screening guidelines, weekly meetings were held among the modeling team, representatives from the WHO secretariat and representatives from the systematic review and costing teams. Regular meetings were also held among members of the Guidelines Development Group members and the systematic review, modeling and costing teams to discuss the priority management algorithms.

The modeled evaluation was performed over a three-stage process (Supplementary Fig. 1). In the first stage, we evaluated the benefits and harms (using pre-cancer treatments as a proxy for harms) of the seven priority algorithms, considering various screening ages and frequencies. These results were presented to the Guidelines Development Group in July 2020. In the second stage, we included results on additional adverse obstetric outcomes as a result of pre-cancer treatments as another measure of the harms associated with screening as well as cost-effectiveness outcomes. Modeled evaluations of these algorithms were presented to the Guidelines Development Group in September 2020. The third stage involved a detailed exploration of the optimal management of women after negative triage test and the optimal management of women after treatment for pre-cancer. Modeled evaluations of these alternative management options were presented to the Guidelines Development Group in November 2020.

### Model platform

A validated dynamic model platform, of sexual behavior, HPV transmission, HPV type-specific natural history, cervical screening and vaccination—*Policy1-Cervix*, was used for this evaluation^4,5,7,8,15,17–23,41^. This platform models HPV transmission, type-specific natural history, cervical screening, diagnosis and treatment and has been extensively validated against data from a range of countries. The model simulates HPV infection, which can persist and/or progress to cervical intraepithelial neoplasia grades I, II and III (CIN1, CIN2 and CIN3); CIN 3 can then progress to invasive cervical cancer. Progression and regression rates between states are modeled separately for types HPV 16, HPV 18, other high-risk nonavalent-included types (31/33/45/52/58) and other non-nonavalent-included high-risk types (Supplementary Fig. 2). The model platform captures the increased risk of CIN2+ recurrence in successfully treated women (compared to the baseline risk of CIN2+ in the population), as previously described^7^.

The model was used to predict outcomes for each strategy across the lifetime of females aged 10–84 years who turn 30 in 2030 (born 2000) across all 78 LMICs. The *Policy1-Cervix* model was one of three models used by the CCEMC to evaluate the impact of cervical cancer elimination targets in 78 LMICs and was reviewed and endorsed by the WHO Advisory Committee on Immunization and Vaccines-related Implementation Research (IVIR-AC) for the use in CCEMC modeling of elimination for the WHO^4,5^. *Policy1-Cervix* was also used to predict the timeline to elimination of cervical cancer for 181 countries^22^, for the United States^21^ and for Australia^15^. It has been used for a range of government-commissioned studies on behalf of national cervical screening programs in Australia, New Zealand and England. Some specific examples of this include the effectiveness modeling and economic evaluation of cervical screening for both unvaccinated cohorts and cohorts offered vaccination, as part of the renewal of the cervical screening program in Australia^7^, as well as similar screening policy evaluations for New Zealand^8^ and England^17^. It has also been used to provide estimates of resource utilization and disease impacts during the transition from cytology to HPV screening in Australia and New Zealand^42–44^, to inform clinical management guidelines in Australia^45^ and to evaluate the impact of adopting self-collected HPV testing in Australia^19^. It was previously extensively validated and used to evaluate changes to the screening interval in Australia and the United Kingdom^17,46^, the role of alternative technologies for screening in Australia, New Zealand and England^17,47–49^, the role of HPV testing for the follow-up management of women treated for cervical abnormalities^50^, the cost-effectiveness of alternative screening strategies and combined screening and vaccination approaches in China^51,52^, the impact of HPV vaccine hesitancy in Japan^20^ and the cost-effectiveness of primary HPV testing and the potential for elimination in Malaysia^26^. The model has also been used to evaluate the impact of HPV vaccination^53^ and the incremental impact of vaccinating males in Australia^18,54^, to evaluate the impact of the nonavalent HPV vaccine in four developed countries^18^ and to assess the cost-effectiveness of the nonavalent HPV vaccine in Australia^41^. Predictions from the dynamic HPV transmission and vaccination model have also been validated against observed declines in HPV prevalence in women aged 18–24 years after the introduction of the quadrivalent vaccine^55^.

Model predictions of age-specific cervical cancer incidence and mortality, the rate of histologically confirmed high-grade lesions per 1,000 women screened and overall screening participation rates were previously validated against national data from Australia, England and New Zealand^8,17,56^ after taking into account local age-specific screening behavior obtained by analysis of screening registry data. *Policy1-Cervix* has also been used in conjunction with a model of fertility to estimate the impact of vaccination and screening changes on adverse pregnancy outcomes^57^, and ethnicity-specific models have been developed for New Zealand^58^. More details on the model structure, previous applications and calibration documentations for selected countries can be found on our Policy1 website^59^.

#### Model calibration

The model calibration to 78 LMICs was described in detail previously^4,5^ and is summarized below. As there are no reliable nationally or regionally representative data on sexual behavior across all LMICs, we relied on GLOBOCAN2018 estimates of age-specific cervical cancer incidence to inform the age-specific rate of acquiring new HPV infections, an approach that was taken in previous evaluations and by other modeling teams^4,5^. Institute for Health Metrics and Evaluation (IHME) sub-regional-level estimates for the stage distribution of invasive cervical cancer at diagnosis, and data on 5-year and 10-year survival rates, were derived from systematic reviews done by the WHO based on peer-reviewed publications and national reports, including cancer control plans, cross-referenced to data from IARC cancer registries and stratified by levels of cancer treatment access^4,5^. We used 2018 data for radiotherapy access and availability of external beam radiation therapy and personnel provided by the International Atomic Energy Agency’s Directory of Radiotherapy Centres (DIRAC) as a measure of treatment access in a country. These data were then combined to derive estimates of 5-year and 10-year stage-specific survival for each country. We used these data as inputs to the model and additionally applied a quality factor into the estimated survival assumptions to calibrate against GLOBOCAN2018 estimates for cervical cancer mortality. Calibration to GLOBOCAN2018 estimates was previously reported for CCEMC models^4,5^ and is specifically reported here for the *Policy1-Cervix* model in Supplementary Fig. 3.

The quality of data informing the burden of disease across the 78 LMICs is limited; for instance, the cancer incidence and mortality targets from GLOBOCAN 2018 represent estimates based on neighboring countries for many LMICs, whereas cancer incidence and mortality rates for high-income countries is mostly based on nationally representative cancer registration data. Additionally, the inputs on cancer treatment access were based on radiotherapy machine density as a surrogate for treatment access across all cancer stages. For this reason, we note that it is important to use a model that has been extensively validated across settings with sufficient data to inform unobservable features, such as underlying natural history rates. The *Policy1-Cervix* model is well suited for such an evaluation as it has been extensively validated against data from a range of countries, including settings in which a substantial amount of data were available, including type-specific HPV prevalence, rates of abnormal tests, rates of histologically detected high-grade disease, type-specific cervical cancer incidence and cervical cancer mortality, as described in the previous section.

Our previous two papers on the impact of elimination strategies on cervical cancer incidence and mortality^4,5^ took a comparative modeling approach using three well-established modelling platforms: *Policy1-Cervix*, Harvard and Laval. All three models produced a good fit to GLOBOCAN estimates through the calibration phase and predicted similar elimination timing, reductions in cervical cancer incidence and reductions in cervical cancer mortality when modeling the impact of elimination strategies across 78 LMICs^4,5^ as well as elimination outcomes for high-income countries^60^. This concordance across three independently developed models provides confidence that all three models are accurately capturing the natural history of cervical cancer and impacts of cervical cancer prevention. The *Policy1-Cervix* platform was reviewed and endorsed by the WHO IVIR-AC for the use in modeling elimination targets across the 78 LMICs for the WHO. A list of each of the 78 countries included, along with their GDP per capita, is provided in Table 5. Reporting is performed according to HPV-FRAME standards for models evaluating HPV vaccination and cervical screening^61^ (Supplementary Table 2).

### Model of obstetric complications

To evaluate adverse obstetric outcomes due to pre-cancer treatment, we developed a Monte Carlo individual-based simulation model that incorporates country-specific and age-specific fertility rates, as well as pre-cancer treatment outcomes by mode of treatment, and explicitly models additional pre-term delivery events as a result of ablation and excisional treatments at an average level across all 78 LMICs. This model was adapted from a module that has previously been used to simulate adverse obstetric outcomes after cervical screening in high-income countries^57^. Outputs from the *Policy1-Cervix* platform for first pre-cancer treatment event by age and treatment type (ablation versus excisional) are input into the Monte Carlo model. This model then evaluates the number of pre-term delivery events as a direct result of pre-cancer treatment. Combining systematic review evidence on the risk of pre-term delivery after excision (excision versus no treatment: 11.2% versus 5.5%, RR = 1.87, 95% CI: 1.64–2.12)^30^ with a detailed model of cervical cancer screening and pre-cancer treatment for Australia^57^, estimated pre-term delivery events for Australia^57^ and Australian fertility data, we estimated that women with a history of excisional treatment have an excess probability of pre-term delivery of 4.8% for each subsequent pregnancy. Systematic reviews indicate that the risk of pre-term delivery after ablation is lower than that after excision (ablation versus no treatment: 7.7% versus 4.6%, RR = 1.35, 95% CI: 1.20–1.52)^30^. We then estimated that the additional probability of pre-term delivery per pregnancy in women with a history of ablation without excision is (1.35−1) / (1.87−1) × 4.8% = 1.9%. We obtained national age-specific fertility rates for each of the 78 LMICs from the United Nations (2019)^62^ and performed a population-weighted average to generate fertility rates for all 78 LMICs. We conservatively assumed that multiple treatments of the same type do not generate any additional risk of adverse pregnancy outcomes. In sensitivity analysis, we also considered a scenario in which ablative treatments did not increase the probability of pre-term deliveries for subsequent pregnancies.

### Screening strategies

We considered the benefits, harms and cost-effectiveness of seven priority screening algorithms as identified by the Guidelines Development Group compared to no-screening: primary VIA, primary cytology with HPV DNA triage (ASC-US referral), primary HPV DNA without triage (assessment of eligibility for ablative treatment), primary HPV DNA with HPV16/18 triage, VIA triage, cytology triage and colposcopy triage. Screening ages and frequencies considered for this analysis are shown in Supplementary Table 1. Detailed management for each of these screening scenarios, including downstream management for women in follow-up, at colposcopy and after pre-cancer treatment, are described in Supplementary Fig. 4. Variations in age ranges and frequencies considered generate a total of 19 scenarios.

### Test performance

An updated systematic review was conducted to inform the Guidelines Development Group on cross-sectional sensitivity and specificity of a range of screening and triage tests for both the general population of women and women living with HIV. This updated review was then used to inform model inputs for test performance. The updated review did not include primary HPV or primary cytology performance, and so published systematic review evidence was used for these tests. The sensitivity and specificity reported for all tests evaluated are shown in Supplementary Table 3.

Results from the published systematic reviews indicate a sensitivity to CIN2+ of 94% for primary HPV DNA testing, and, based on uncertainty ranges reported in the literature, we consider a range of 88−96% sensitivity to CIN2+ in sensitivity analysis. These studies include a range of validated HPV DNA testing assays, which may target slightly different groups of HPV types, although they overlap on the most oncogenic ones^32^. Studies indicated a sensitivity of 70% for CIN2+ for primary cytology testing, and, based on uncertainty ranges reported in the literature across both conventional and liquid-based cytology, we consider a range of 46.8−80% in sensitivity analysis.

Test performance for primary VIA was based on a combination of evidence from cross-sectional studies and larger-scale population-level longitudinal studies. A 2022 study found that, for women living with HIV, when considering studies with more than 95% histological verification of disease involving (totaling 1,700 women), VIA had a low sensitivity to detect CIN2+ of 56% (95% CI: 45.4−66.1%)^11^, and it was noted that there was substantial heterogeneity in the performance of VIA. Additionally, many cross-sectional studies, which informed the systematic reviews on test performance, were short-term pilot studies with intensive training of staff, and the smaller scale and short duration of the studies means that relatively favorable findings may not be translatable for longer-term population-wide settings.

Several VIA-based screening experiences have been documented in LMICs. Longitudinal studies of large population-based implementation of VIA have been performed in India. In 2014, results from a large community-based randomized controlled trial in India (1998−2011) reported cervical cancer incidence and mortality after four VIA screening rounds over 12 years^9^. This study included 70,000 women participating in each of two screening arms—no screening and primary VIA testing—resulting in a total study size of more than 140,000 women followed-up over 12 years. Results indicated that there was no significant reduction in cervical cancer incidence in the VIA arm, and a mortality reduction of 31% (RR = 0.69, 95% CI: 0.54−0.88)^9^, implying some cancer downstaging but low sensitivity for detecting high-grade lesions that could progress to cancer. In 2009, another randomized controlled trial assessed the impact of a single round of HPV, VIA and cytologic testing on cervical cancer incidence and mortality in Osmanabad District, India. It included over 30,000 women in the VIA screening arm and found that women offered VIA testing had no significant reduction in stage II+ incidence or mortality after one round of screening (8 years of follow-up after the screening event)^10^.

Although these larger population-wide studies could not be used to directly obtain sensitivity and specificity of the VIA test, they are of substantial size, are conducted over longer periods of time and are critical studies to consider when deciding on the population-level performance of VIA testing. We performed a modeled simulation of both trials to identify what test performance assumptions of VIA would generate the reductions in incidence and mortality observed. We found that sensitivity rates to CIN3+ of 40% or lower produced the most accurate predictions, and test sensitivity for women with cervical cancer of 60% produced the most accurate reductions in cervical cancer mortality (details not shown). Based on the availability of evidence from cross-sectional studies and larger-scale population-level longitudinal studies, the Guidelines Development Group agreed that, for VIA, we would assume 40% sensitivity to CIN2+ for the base case analysis, and, given that there was some evidence of improved mortality from VIA screening, we additionally assumed that VIA can detect cervical cancer with 60% sensitivity. We also consider 60% sensitivity to CIN2+ and 88% sensitivity to cancer as a favorable upper bound (‘high sens’).

The sensitivity and specificity were used to generate detailed test probability matrices for the base case analysis, which represent the probability of certain test results given a woman’s true underlying health state for the three tests considered: VIA, cytology and HPV (Supplementary Tables 3 and 4). These test matrices, when evaluated for screening programs, produce a range of potential sensitivity and specificity values that are dependent on the underlying disease of the population. Therefore, we present a range of model-predicted sensitivity and specificity considering variation in disease rates across six geographical regions (East Asia & Pacific, Europe & Central Asia, Latin America & Caribbean, North Africa & the Middle East, South Asia and Sub-Saharan Africa) and across different age groups within each region. Specifically, we used disease state outputs from the models for each of the six geographical regions and across three age groups per region: 25−34 years, 35−44 years and 45−54 years. The calculated sensitivity and specificity are presented as the range across the 6 × 3 = 18 combinations of regions and age groups for each test. For cytology and HPV-based screening, the model ranges for sensitivity and specificity compare well with the systematic review data used to inform the inputs.

For sensitivity analysis, we considered the impact of the upper and lower sensitivity performance for each of the primary screening tests (VIA, cytology and HPV) on incidence and mortality outcomes. When modeling the upper and lower ranges for test performance, we do not explicitly generate updated test probability matrices for these test performance assumptions. Rather, we scale the predicted reduction in incidence and mortality based on the relative difference in sensitivity of each range compared to base case.

We assume that the probability that a woman is eligible for same-day ablation is dependent on the underlying disease state. For women with <CIN2, 98% are eligible for same-day ablation, and, for women with CIN2+, 85% would be considered eligible for same-day ablation (Supplementary Fig. 4).

For successfully delivered ablative pre-cancer treatment, we assumed that 81% of CIN2/3 will be successfully treated (that is, lesion is completely removed), based on a review of the literature^63–65^. For excisional treatment, we assumed 93.6% treatment success for excisional treatment for CIN2/3, based on international literature reviews^66–69^. For both ablation and excisional treatments, we assume that, of the women who are successfully treated, 85% will also clear the underlying infection, whereas 15% will remain HPV infected^70^.

At colposcopy, we assumed that women receive an abnormal colposcopy result at a rate of 50% if they have <CIN1, 77% if they have a productive HPV infection with CIN1 and 88% if they have CIN2+. This was informed from a large colposcopy dataset (over 21,000 colposcopies) supplied by the Royal Women’s Hospital in Victoria, Australia^46,49^. We assumed that biopsy and endocervical curettage (ECC) are 100% accurate at identifying underlying disease. We assume that women will receive a result of type 3 TZ at colposcopy based on their age, with women aged younger than 25 years having a 2.01% chance of type 3 TZ; women aged 25–29 years having a 2.78% chance; women aged 30–34 years having a 6.03% chance; women aged 35–39 years having a 7.5% chance; women aged 40–44 years having a 12.56% chance; women aged 45–49 years having a 19.48% chance; women aged 50–54 years having a 30.98% chance; and women aged ≥55 years having a 45.66% chance. The rationale for this was described previously^7^.

For invasive cancers, all scenarios assumed invasive cervical cancer clinical staging according to the International Federation of Gynecology and Obstetrics (FIGO) system. In the comparator (‘no screening’), a proportion of clinically/symptomatically detected cervical cancers is assumed to be actively treated, with proportions varying by country (averaging up to 33% of symptomatically detected cases in LMICs receiving cancer treatment), as described in our previous evaluation^5^. Stage distribution at detection was provided by the WHO for the earlier elimination analysis performed as part of the CCEMC^5^.

All screening scenarios assumed that 90% of screen-detected cancers would receive adequate treatment and care, and, therefore, we assumed improved survival for screen-detected cancers. The relative survival for screen-detected cervical cancer compared to symptomatically detected cervical cancer is additionally assumed to be scaled up by 1.15 for localized disease and by 1.17 for regional/distant disease, regardless of whether the woman received adequate treatment and care^71–73^, as described previously^5^.

### Screening adherence

In this normative analysis across countries, we made favorable assumptions about screening and follow-up attendance to predict the potential impact and cost-effectiveness of high-coverage cervical screening in LMICs. In our discussions with the Guidelines Development Group, we aimed to choose assumptions that represented the ‘realistic best-case scenario’, understanding that, especially at the inception of new programs, participation is unlikely to be this high in all settings. For the base case analysis, the targets for screening coverage by 2030 were based on the WHO’s Global Strategy toward the elimination of cervical cancer, which was endorsed by all Member States in 2020 and therefore we assumed that 70% of women attend each routine screening visit but that 10% would be never-screeners (so the 70% are selected from the 90% of ever-screeners), and we considered lower screening compliance rates of 50% in sensitivity analysis. We made the favorable assumption that women referred for follow-up or treatment would attend at 90% adherence if the follow-up was to occur on a later day. If same-day treatment could be offered—for instance, primary HPV with VIA triage or primary HPV without triage—we assumed that a PoC HPV test was used 50% of the time and that 100% compliance with follow-up is achieved when the PoC test is used. This results in an average of 95% of women complying with same-day treatment after primary HPV with VIA triage or primary HPV without triage. We assumed that same-day test and treatment would be available for all primary VIA scenarios and, therefore, made the favorable assumption that 100% adherence would be achieved in women eligible for same-day treatment after primary VIA. We assumed, in consultation with the Guidelines Development Group, that women who do not comply with follow-up or treatment are lost for that screening round. These women have the option to attend their next screening event (at 70% compliance) or will no longer return if they are beyond the screening age recommendation. Clearly, these assumptions reflect very high levels of participation and compliance, which has not yet been achieved in many LMICs. However, the intent of this modeling evaluation was to provide information on the maximally achievable outcomes from implementing screen, triage and treat strategies at scale (‘normative’ modeling evaluation).

We assumed that 90% of screen-detected cervical cancer cases would receive adequate treatment; however, access to cancer treatment for symptomatically detected cancers would remain unchanged from the status quo (rates vary by country^5^). In sensitivity analysis, we considered a favorable scenario in which 90% of both screen-detected and symptomatically detected cervical cancers received adequate treatment and also a less favorable scenario in which both women symptomatically-detected and screen-detected for cancer receive status-quo access rates for cancer treatment access.

### Outcomes assessed

For each strategy, we reported on outcomes over the lifetime of unvaccinated women who would turn 30 in 2030—the first cohort to be fully impacted by scale-up of cervical screening to 70% coverage by 2030. Outcomes assessed include the lifetime number of cervical cancer cases and deaths and age-standardized incidence and mortality rates as a measure of the benefits, with standardization using the estimates of the 2015 World Female Population from the UN World Population Projections for ages 0–99 years. We assessed the number of pre-cancer treatments needed to prevent a cervical cancer death (NNT) and pre-term delivery events due directly to pre-cancer treatment (‘additional pre-term delivery events’) as a measure of the harms associated with screening. We also report on resource utilization events, including the lifetime number of VIA, cytology and HPV tests, ablation and excisional treatment events and colposcopy and biopsy events. We report on the cost and cost-effectiveness of each strategy as a cost per HALY saved, assuming 0% discounting for effects and 3% discounting for costs and assuming that discounting starts from age 30. We presented results as a population-weighted average across 78 LMICs, which we refer to as a ‘normative approach’, using the 2015 population structure for population-weighted contribution of each country. We identified strategies that appear on or near the cost-effectiveness frontier as being the strategies with the best balance of costs and effects.

### Populations assessed

We considered the seven priority algorithms over the lifetime of a cohort of 100,000 women who would turn 30 in 2030, the first cohort to experience screening across their whole screening lifetime if screening started in 2030, across 78 LMICs. We included all women living in these settings regardless of HIV status, which is the same approach taken in previous evaluations^4,5^. The 78 LMICs considered were located in six regions according to World Bank definitions: East Asia & Pacific, Europe & Central Asia, Latin America & Caribbean, North Africa & the Middle East, South Asia and Sub-Saharan Africa. Each country included is listed in Supplementary Table 5. All results are presented as a population-weighted average (using population estimates for 2015 from the United Nations^74^) across all 78 LMICs. As done in previous evaluations^4,5,15,22^, we used the World Standard Population (WSP) 2015 for ages 0–99 years to calculate the age-standardized rate (ASR) for cervical cancer incidence and mortality. When estimating the effects across a group of countries, the UN 2015 population estimates are used as the relative weighting for each country.

We considered outcomes in unvaccinated women only. Although vaccination programs may be implemented by this time in many LMICs, females who would be targeted by vaccination programs between 2020 and 2029 will not be screen age eligible until approximately 2040, and, even after this time, many women within the screening age ranges (30–49 years) will be unvaccinated for 1–2 further decades.

The comparator scenario assumes no screening, no vaccination and no scale-up of cervical cancer treatment access. In some LMICs, opportunistic screening is available for some women. Based on a review of the scientific literature, government websites and official documentation^75^, it was found that 9–11% of women in LMICs have ever been screened; however, 72–75% of countries that have offered screening used primary VIA testing, which has low sensitivity in a programmatic setting. Additionally, a high rate of follow-up and adequate treatment and monitoring is required for an effective screening program, which is likely very challenging in these settings. There are also very little detailed data on screening patterns in LMICs at the level of granularity required to model its effect. Information of screening coverage in LMICs is often based on self-reported surveys, which does not provide the full picture of coverage over time, screening approach used and follow-up mechanisms in place for opportunistic screening approaches. For this reason, when considering the comparator for the current evaluation, we assumed that, across the 78 LMICs, no effective programmatic national screening systems are yet in place.

### Discount rates

We reported on cost per HALY saved, assuming that discounting starts from age 30 years. We assumed that a 0% discount rate for effects and a 3% discount for costs was applied in the base case, and a 3% discount rate for both costs and effects was applied in sensitivity analysis, as recommended by the WHO for health economic evaluation of vaccination programs^76^. Although these guidelines were developed for vaccine evaluations, we used them here for this screening evaluation based on expert advice from the WHO.

### Costs and disability weights

A health services/provider perspective was adopted for this analysis, assuming that the government would fund cervical screening programs on the assumption that this would be the preferred mode as countries implementing single-payer healthcare systems have notably had improved equity of access^77^. HPV test costs were informed by CHAI and WHO-CHOICE data, and all other screening-related costs were informed by WHO-CHOICE data^78,79^. Cost data were provided and applied separately for each of the 78 LMICs, but, for simplicity, here we present the population-weighted aggregate cost (‘normative costs’). Normative costs are presented as population-weighted costs across the population of the 78 LMICs. Because these costs are directly incurred at the age at which simulated women are screened, input normative costs are calculated as population-weighted costs, which, in the base case, were done across the population of the 78 LMICs. When weighting input costs across countries, we used the 30–59-year-old female population for each country, where most of the costs are applied and benefits are seen, as opposed to using the entire population (0–99 years) to weight the relative contribution of each country. However, output costs and effects are accrued across all ages modeled (0–84 years). The inclusions for each cost are described in Supplementary Table 6 and in accompanying footnotes for each cost item. We considered the variation of costs and disutilities in probabilistic sensitivity analysis. All costs are in 2019 US$. For supplementary analysis, we also performed regional-level cost-effectiveness analysis (CEA) and used population-weighted costs across each region, as shown in Supplementary Table 7.

Invasive cancer treatment was costed by stage (FIGO staging). Costs are applied only in the instance that a woman receives cancer treatment. Costs for treatment were applied to the first year and included one episode of surgery plus hospitalization and multiple episodes of radiotherapy and chemotherapy. Costs for surveillance were applied from 12 months after diagnosis until death or a maximum of 5 years after diagnosis. Costs are calculated in terms of an aggregate cost by stage at diagnosis that includes costs for the average surveillance time based on survival times for that stage, with weights to indicate how many women would access treatment (treatment access proportion). Costs for palliative care were applied in the year that cervical cancer death occurs (or no costs applied if the woman dies from other causes).

CEA was conducted for the general women population at the level of all 78 LMICs and at the regional level using the 2015 population structure for population-weighted contribution of each country. Findings were presented as cost per HALYs saved. Life-years saved (LYSs) and $ per LYS were explored in sensitivity analysis.

As a reference point for a potential WTP threshold in this population, the population-weighted average GDP per capita for 2019 across the 78 LMICs is US$2,099. Furthermore, 69 of 78 LMICs (89%) have a GDP per capita equal to or above US$530; 52 of 78 LMICs (67%) have a GDP per capita equal to or above US$1,000; and 29 of 78 LMICs (37%) have a GDP per capita equal to or above US$2,000, according to the 2019 World Bank database (Supplementary Table 5)^80^. The use of these macroeconomic thresholds will allow reasonable comparison across countries with very different economic profiles (although it is acknowledged that such thresholds are of limited utility for within-country CEA)^81,82^. We identify strategies that appear on or near the cost-effectiveness frontier as being the strategies with the best balance of costs and effects.

Disability weights for cancer states were estimated by the Global Burden of Disease Study 2010 (ref. ^83^) and were applied to cancer based on stage and time since diagnosis, and they represent disability-adjusted life-years (DALYs). These are shown in Supplementary Table 8. For FIGO stage 1–3, we assumed that a disutility of 0.288 was applied for 12 months after diagnosis and a disutility of 0.049 for each year after the first year, applied for a maximum of 5 years. For FIGO stage 4, we assumed a disutility of 0.451 from diagnosis until 3 months before death or a maximum of 5 years after diagnosis. For women who die from cervical cancer, we assumed that a disutility of 0.54 is applied for the final 3 months of life. As part of sensitivity analysis, we also explored the impact of applying a disutility to women who undergo pre-cancer treatment of 0.01 for 12 months after treatment with ablation, excision or ECC, based on a study into the psychosocial impact of abnormal test findings conducted in high-income settings^84^. These weights were applied to each ECC, ablative treatment and excisional treatment. We also explored outcomes considering life-years only. The base case analysis could be presented as cost per DALY saved; however, the sensitivity analyses cannot be represented by DALYs. We, therefore, present all results as cost per HALY saved but note that the base case results could also be interpreted as cost per DALY saved.

### Calculating costs

Let *m*_*r*_ be the average unit cost of resource *r* across the 78 countries (either as given in WHO data or population weighted over countries), and let ${u}_{a,r}^{i,c}$ be the probability of using that resource *r* at age *a* in country *c* for scenario *i*. The 78-country aggregated average probability of resource *r* being used at age *a* in scenario *i* is:

$${U}_{a,r}^{\,i}=\left({\sum }_{c}{u}_{a,r}^{i,c}\,{n}_{a}^{c}\right)/{N}_{a}$$

The total costs of all resources at each age in scenario *i* is:

$${M}_{a}^{\,i}={L}_{a}^{i}{\sum }_{r}{m}_{r}{U}_{a,r}^{\,i}$$

that is, the sum of the cost-weighted rates of each resource at that age multiplied by the alive population at that age.

We can then divide by ${L}_{10}$ and sum to obtain the discounted costs as usual:

$${{\mathscr{M}}}_{i}=\left(\frac{1}{{L}_{10}}\right)\left(\sum_{a=10}^{29}{M}_{a}^{\,i}+\sum_{a=30}{M}_{a}^{\,i}{\left(1-\delta \right)}^{-\left(a-30\right)}\right)$$

where δ is the discount rate.

### Calculating HALYs

To measure the effects of screening, HALYs were used in base case, and life-years were explored in sensitivity analysis.

To calculate the life-years, we use the country-level incidence and mortality rates combined with the all-cause mortality rates from the UN Population Division webpage^74^.

Let ${p}_{a}^{c}$ be the probability of dying from all causes at age *a* in country *c*, and let ${\gamma }_{a}^{i,c}$ be the probability of dying from cervical cancer at age *a* in scenario *i* in country *c*. We define the probability of dying from causes other than cervical cancer at age *a* in country *c* to be:

$${q}_{a}^{c}={p}_{a}^{c}-{\gamma }_{a}^{0,c}$$

We use United Nations population estimates and projections of each country for 2015, defined as ${n}_{a}^{c}$ for age *a* in country *c*. The age-specific population over all countries is then ${N}_{a}=\sum _{c}{n}_{a}^{c}$.

Then, we obtain the average 78-countries probability of dying of cervical cancer by taking the population-weighted average: ${\varGamma }_{a}^{i}=(\sum _{c}{n}_{a}^{c}{\gamma }_{a}^{i,c})/{N}_{a}$

Similarly, we obtain the 78-countries average of other-cause mortality: ${Q}_{a}=(\sum _{c}{n}_{a}^{c}{q}_{a}^{c})/{N}_{a}$

We assume that the population alive at age 10 years across the 78 countries is ${L}_{10}={N}_{10}$.

From this, we can incrementally calculate the number alive at subsequent ages in a hypothetical global cohort: ${L}_{a+1}^{i}={L}_{a}^{i}(1-{Q}_{a}-{\varGamma }_{a}^{i})$

We can then divide each of the number alive by ${L}_{10}$ and sum to obtain the discounted life-years as usual:

$${{\mathscr{L}}}_{i}=\left(\frac{1}{{L}_{10}}\right)\left(\mathop{\sum}\limits_{a=10}^{29}{L}_{a}^{i}+\mathop{\sum}\limits_{a=30}{L}_{a}^{i}{\left(1-\delta \right)}^{-\left(a-30\right)}\right)$$

where δ is the discount rate.

To calculate HALYs, we used the disability weights shown in Table 5. Let ${A}_{s}^{c}$ be the wrapped-up disutility of being diagnosed (that is, the cervical cancer incidence) and ongoing surveillance with FIGO stage *s* in country *c*.

Let *B* be the disutility of palliative care (cervical cancer mortality), which is the same for all countries and stages.

Let ${\theta }_{a,s}^{\,i,c}$ be the probability of being diagnosed with FIGO stage *s* cervical cancer at age *a* in country *c* for scenario *i*.

Then, ${\varTheta }_{a,s}^{i}=\sum _{c}{{{A}_{s}^{c}n}_{a}^{c}\theta }_{a,s}^{i,c}$ is the average disutility of diagnoses and ongoing treatments of each stage *s* of 78 countries for age *a* and scenario *i*.

We then estimate each HALY as: ${H}_{a}^{\,i}={L}_{a}^{i}\left(1\right.-{\sum }_{s=1}^{4}{\varTheta }_{a,s}^{i}-B{\varGamma }_{a}^{i}$

We can then divide by ${L}_{10}$ and sum to obtain the discounted HALYS as usual:

$${{\mathscr{H}}}_{i}=\left(\frac{1}{{L}_{10}}\right)\left(\mathop{\sum}\limits_{a=10}^{29}{H}_{a}^{\,i}+\mathop{\sum}\limits_{a=30}{H}_{a}^{\,i}{\left(1-\delta \right)}^{-\left(a-30\right)}\right)$$

where δ is the discount rate.

### Calculating ICERs

Putting all of the above together, the ICER between two scenarios *i* and *j* at the 78 aggregated countries is then calculated the usual way:

$${ICE}{R}_{{ij}}=\frac{{{\mathscr{M}}}_{i}-{{\mathscr{M}}}_{j}}{{{\mathscr{H}}}_{i}-{{\mathscr{H}}}_{j}}$$

### Supplementary analysis—alternative follow-up management

#### Management of HPV positive and triage negative

For the base case, we assumed that women who tested HPV positive and triage negative would return in 12 months for an HPV test; if negative at this visit, women are then referred for their next routine screening visit or discharged from screening. As a supplementary analysis, we considered two alternative management options for this group based on discussions with the Guidelines Development Group: one was a less aggressive management option in which triage-negative women return in 24 months for the follow-up HPV test (assuming 10% loss to follow-up for the return visit at 24 months but also considering a supplementary analysis of 30% loss to follow-up), and another was a more aggressive management option in which women return at both 12 months and 24 months, with 10% loss to follow-up assumed for each visit; in this more aggressive scenario, women are returned to routine screening or discharged from screening after testing negative at both visits.

#### Management of women after treatment for pre-cancer (and who did not have CIN3 detected by histology)

For the base case, we assumed that women who have been treated for cervical pre-cancer and did not have a histological diagnosis of CIN3 would return in 12 months for an HPV test and are returned to routine screening (or discharged if outside of the age range) if negative at this visit. In the supplementary analysis, we considered alternative management scenarios as informed by discussion with the Guidelines Development Group. One was the option in which these women would return in 24 months for an HPV test and assumed a 30% loss to follow-up at this extended timeframe. The other was an option in which these women return at 12 months for an HPV and cytology co-test, with a 10% loss to follow-up assumed at this visit; women are returned to routine screening (or discharged if outside of the age range) after testing negative with both tests.

### Sensitivity analysis

A range of sensitivity analyses were considered. A lower screening adherence scenario, in which we assumed 50% adherence with routine attendance (30% of women never attend, 50% selected from the pool of ever-screeners) and 75% for adherence with treatment or follow-up visits (100% for same-day eligibility), was explored for all screening approaches. We also performed sensitivity analysis on primary test performance assumptions, including a lower-bound CIN2+ sensitivity assumption of 30% for VIA, 46.8% for cytology and 88% for HPV testing and an upper-bound CIN2+ sensitivity assumption of 60% for VIA, 80% for cytology and 95.7% for HPV. We considered a scenario in which 90% of symptomatically detected cancers received adequate treatment in addition to the screen-detected cases and a scenario in which both symptomatic and screen-detected cancers received treatment at current access rates (33% across all 78 LMICs). We also performed one-way sensitivity analysis assuming a 3% discount rate for both costs and effects and considering life-years instead of HALYs.

PSA was also performed to explore uncertainties in costs and disutilities. We generated 10,000 cost and disutility parameter sets based on the upper and lower ranges for each parameter as described in Supplementary Table 6 (these ranges were discussed with the WHO Guidelines Development Group). To generate the sets, we divided cost values into five independent groups of variables, namely (1) cancer diagnosis, staging and treatment costs; (2) pre-cancer treatment costs; (3) HPV test costs; (4) VIA test costs; and (5) cytology test costs, and we generated 10,000 samples with Latin hypercube sampling. The disutilities formed a single set that varied together from 0% (no disutilities) to 100% (the current disutility assumptions). Acceptability curves were generated for a range of WTP values from US$100 to US$2,000 per HALY saved.

### HPV-FRAME reporting standard checklist

The checklist against HPV-FRAME criteria is shown in Supplementary Table 2. The checklist includes core reporting standard, reporting standard for model of HPV vaccination, model of integrated HPV vaccination and cervical screening and model for LMICs, according to Canfell et al.^61^.

### Statistical analysis

This study does not include a statistical analysis component. When simulating women in *Policy1-Cervix*, we simulated 30 million women from birth to age 85 years across 78 LMICs for each of the six regions that we modeled, as described in our methods. We chose this many women to ensure that the outcomes for life-years, cancer incidence and mortality were smooth and that randomness in the simulation did not impact outputs.

### Ethics and inclusion

This paper is one of a pair of papers to inform the updated WHO 2021 guidelines for screening and pre-cancer treatment for cervical cancer prevention—one for the general population and the current paper for women living with HIV. This research was conducted in close collaboration with the WHO Guidelines Development Group for Screening and Treatment to Prevent Cervical Cancer, which comprises a range of scientists, healthcare providers, implementers, ministry of health representatives, systematic reviewers, program implementation experts and representatives from civil society. The Guidelines Development Group contained members from five WHO regions (AFRO, SEARO, WPRO, EURO and EMRO), many from LMICs. Their names are listed in the annex of the WHO guidelines (https://www.who.int/publications/i/item/9789240030824), and, using the GRADE framework and the WHO Handbook for Guideline Development, cervical screening options, with a focus on LMICs, including countries with high HIV prevalence, were assessed.

### Reporting summary

Further information on research design is available in the Nature Portfolio Reporting Summary linked to this article.

## Online content

Any methods, additional references, Nature Portfolio reporting summaries, source data, extended data, supplementary information, acknowledgements, peer review information; details of author contributions and competing interests; and statements of data and code availability are available at 10.1038/s41591-023-02600-4.

### Supplementary information

Supplementary Information Contains supplementary figures and tables for the Methods

Reporting Summary

## Data Availability

Data on cost inputs (provided separately for the six regions), test performance, health economic parameters, screening algorithms and compliance assumptions relevant to this specific evaluation are described in the Methods. Data on country-specific GDP per capita are also provided in the Methods. Demographic data and data informing the calibration to cervical cancer incidence and mortality across the 78 LMICs and separately for the six regions are described in the Methods and in more detail in previous publications^4,5^.

## Code availability

The model used for this evaluation, *Policy1-Cervix*, is a well-established model platform spanning multiple software programs and related tools, which has been developed over a period of 20 years. The code for these software programs is propriety property. These software programs, modules and tools consist of multiple versions for use in different contexts, and their accurate and appropriate use requires considerable supervised training. For these reasons, the code cannot be provided universally by the authors at this time. We will consider collaborative opportunities harnessing the code, and interested parties are encouraged to contact the Daffodil Centre at info@daffodilcentre.org. Discussions can be held with interested parties within 4–8 weeks of the request and plans made for work put in place after that time, if successful. Appropriately resourced supervision will be provided by Daffodil Centre staff.
