# Supplementary material for: Benefits, harms and cost-effectiveness of cervical screening, triage and treatment strategies for women in the general population
Source: Nat Med. 2023 Dec 12;29(12):3050–8. doi: 10.1038/s41591-023-02600-4 (PMC10719104; doi:10.1038/s41591-023-02600-4)
Supplement: Supplementary file 1 — Contains supplementary figures and tables for the Methods [file 41591_2023_2600_MOESM1_ESM.pdf]

nature medicine

Article

https://doi.org/10.1038/s41591-023-02600-4

Benefits, harms and cost-effectiveness of cervical screening, triage and treatment strategies for women in the general population

In the format provided by the authors and unedited

Supplementary Figure 1: Schematic showing how the systematic review data, cost data and screening algorithms interact with the Policy1-Cervix platform (a). Three stage process for the modelled evaluation to inform the updated WHO cervical screening guidelines (b)

(a)

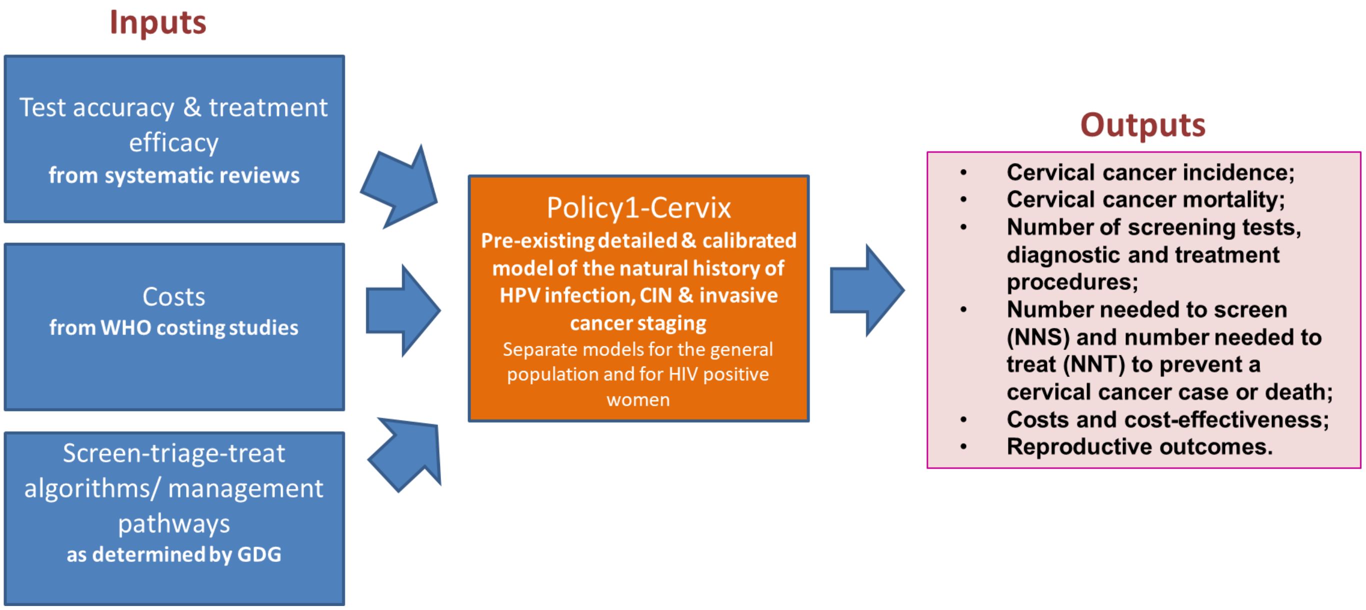

(b)

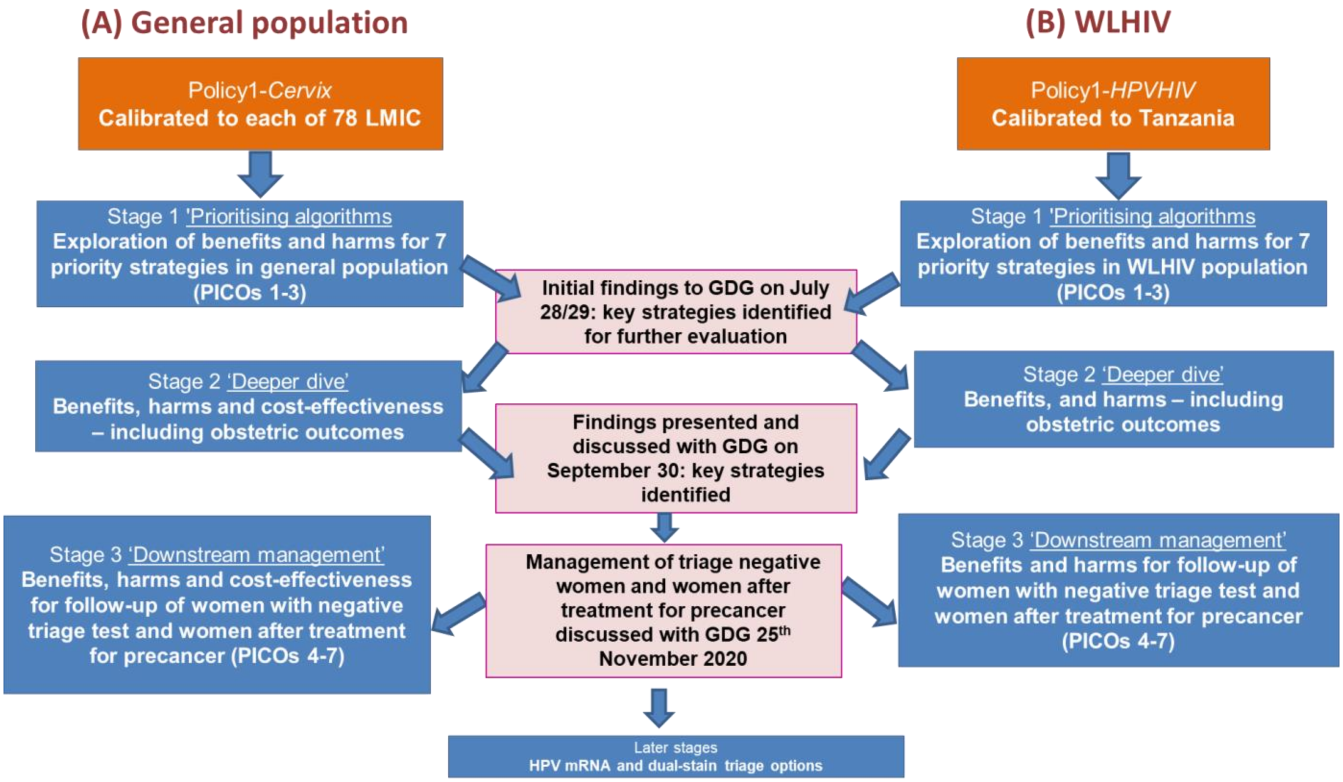
(A) General population
(B) WLHIV
Policy1-Cervix
Calibrated to each of 78 LMIC
Policy1-HPVHIV
Calibrated to Tanzania
Stage 1 'Prioritising algorithms
Exploration of benefits and harms for 7 priority strategies in general population (PICOs 1-3)
Stage 1 'Prioritising algorithms
Exploration of benefits and harms for 7 priority strategies in WLHIV population (PICOs 1-3)
Initial findings to GDG on July 28/29: key strategies identified for further evaluation
Stage 2 'Deeper dive'
Benefits, harms and cost-effectiveness – including obstetric outcomes
Stage 2 'Deeper dive'
Benefits, and harms – including obstetric outcomes
Findings presented and discussed with GDG on September 30: key strategies identified
Stage 3 'Downstream management'
Benefits, harms and cost-effectiveness for follow-up of women with negative triage test and women after treatment for precancer (PICOs 4-7)
Management of triage negative women and women after treatment for precancer discussed with GDG 25th November 2020
Stage 3 'Downstream management'
Benefits and harms for follow-up of women with negative triage test and women after treatment for precancer (PICOs 4-7)
Later stages
HPV mRNA and dual-stain triage options

Supplementary Figure 2: Policy1-Cervix model platform

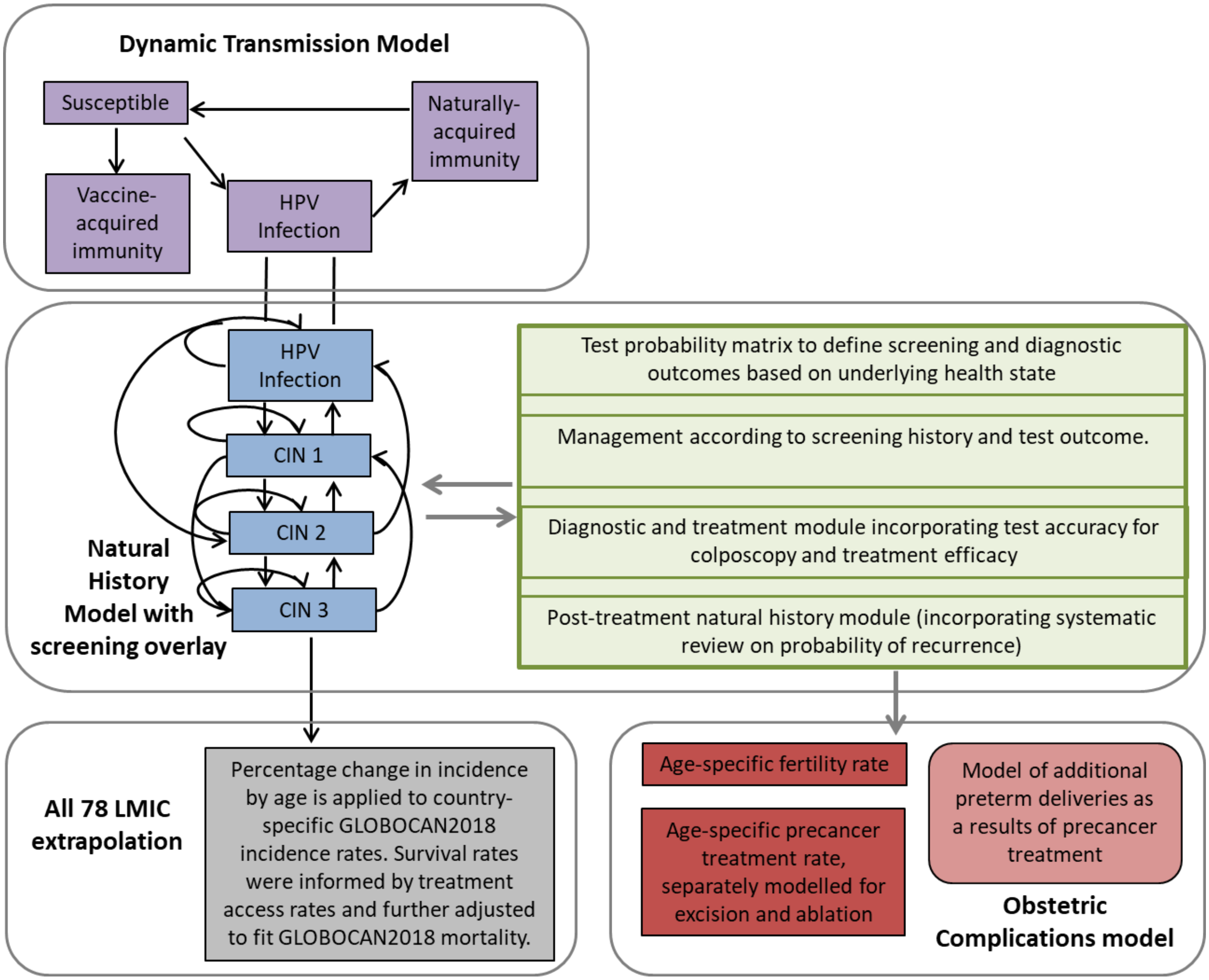

Supplementary Figure 3: calibration curves across All 78 LMICs and across the regions

(a) All-78 LMICs

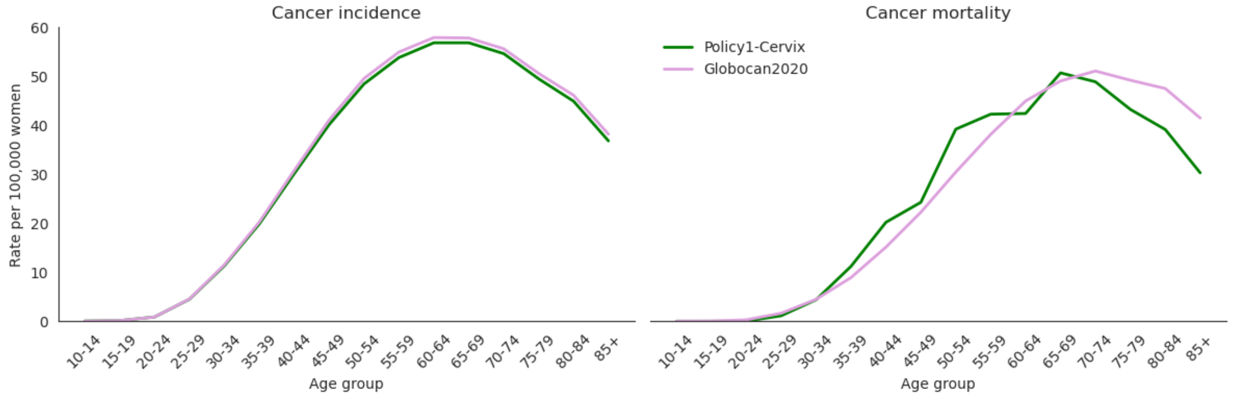

(b) East Asia & Pacific

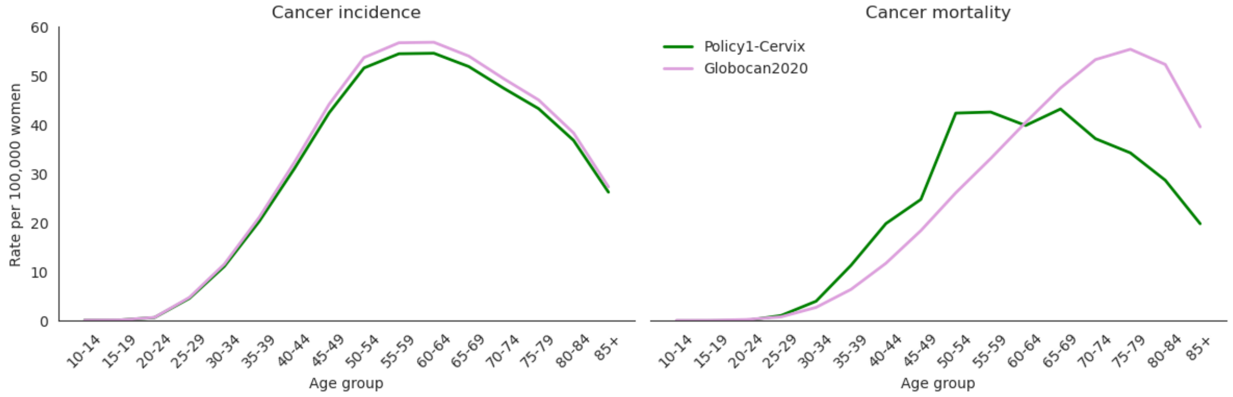

(c) Europe & Central Asia

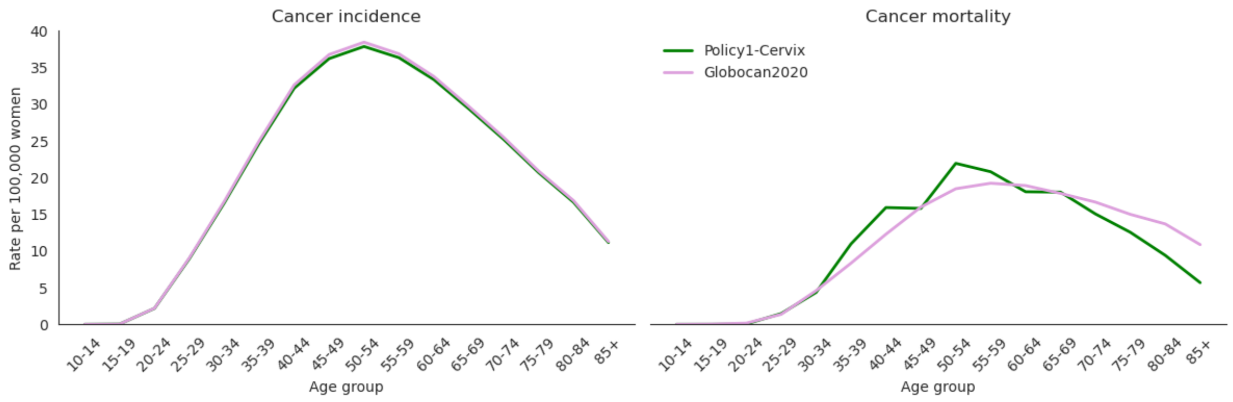

(d) Latin America & Caribbean

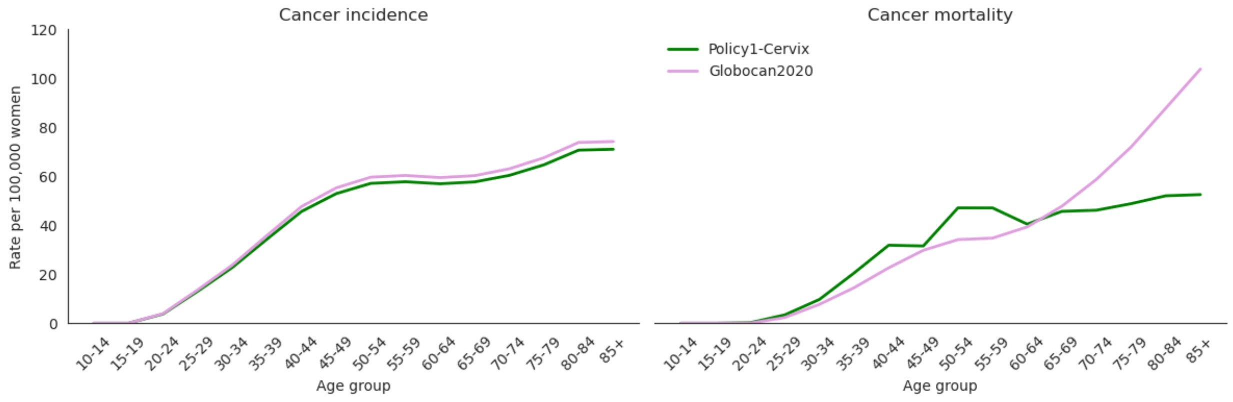

(e) Middle East & North Africa

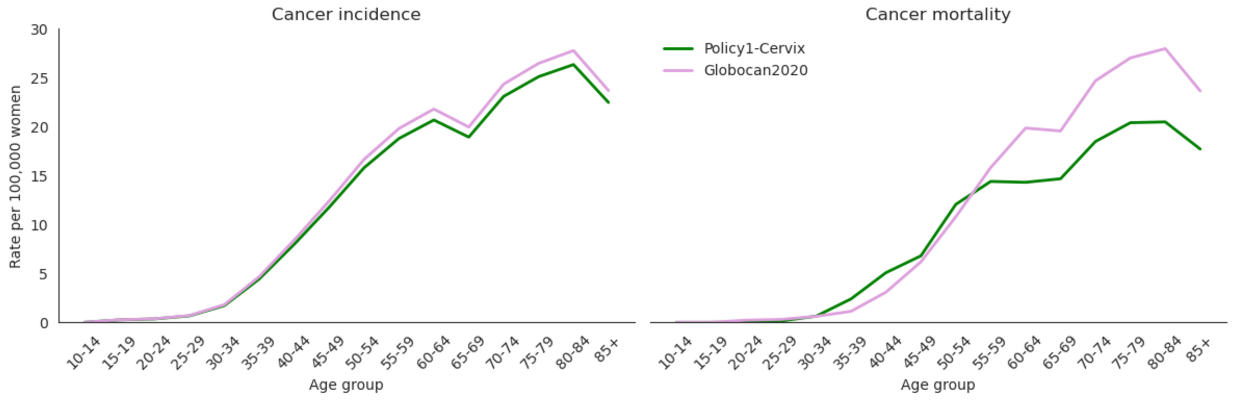

(f) South Asia

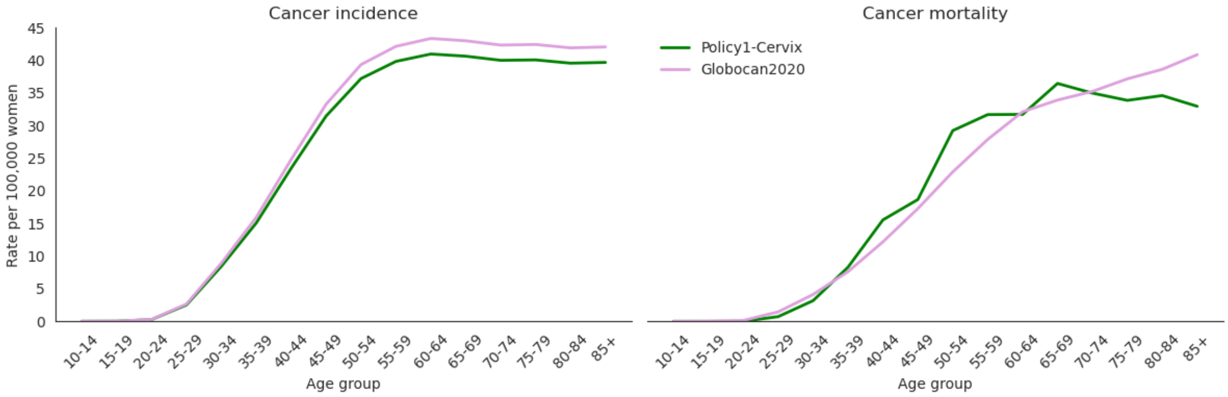

(g) Sub-Saharan Africa

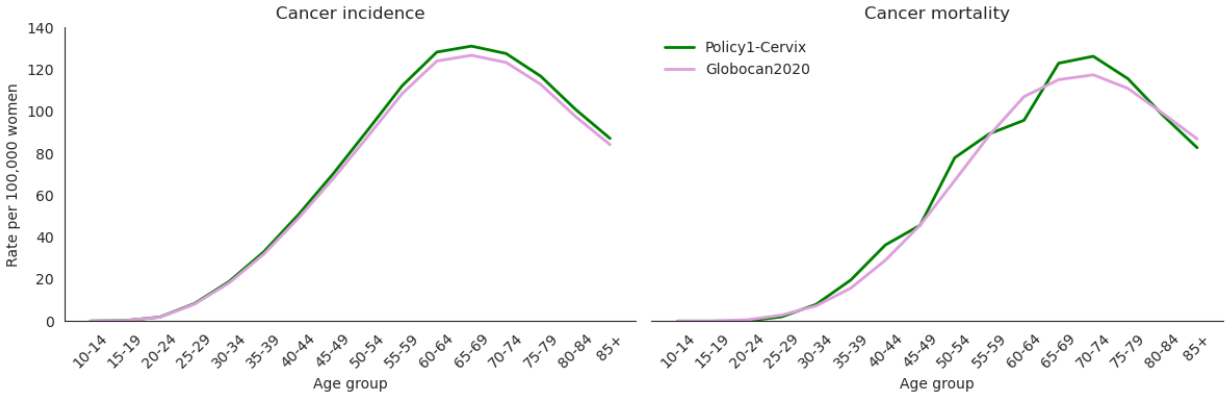

Supplementary Figure 4: Priority screening algorithms

(a) Primary VIA

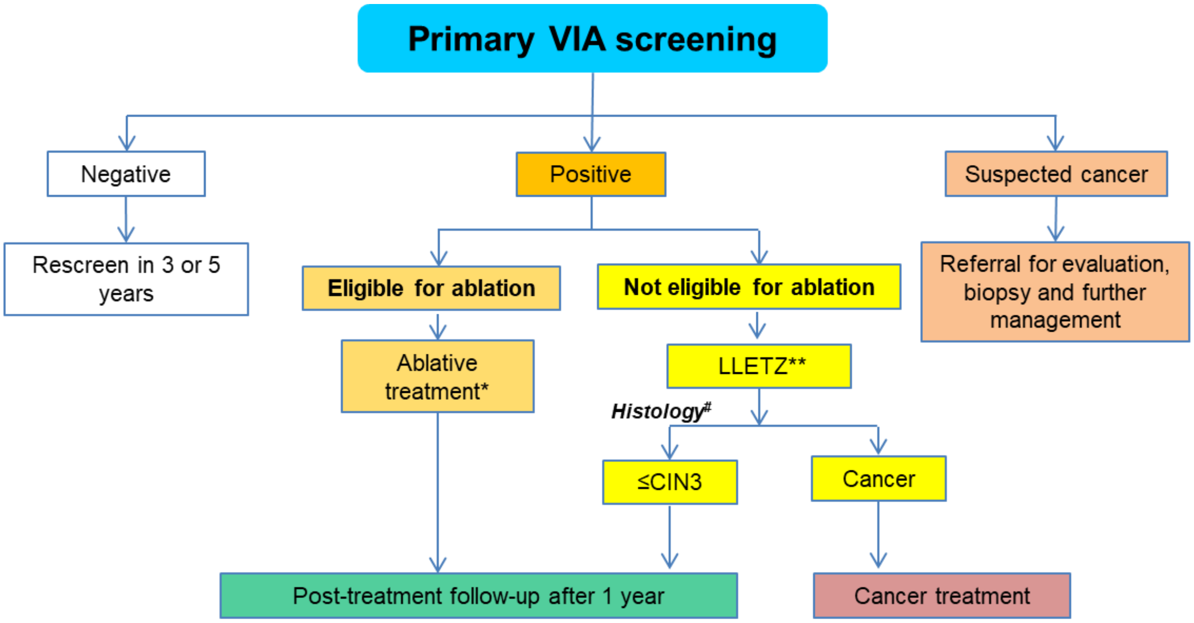

(b) Primary cytology, HPV triage for ASCUS

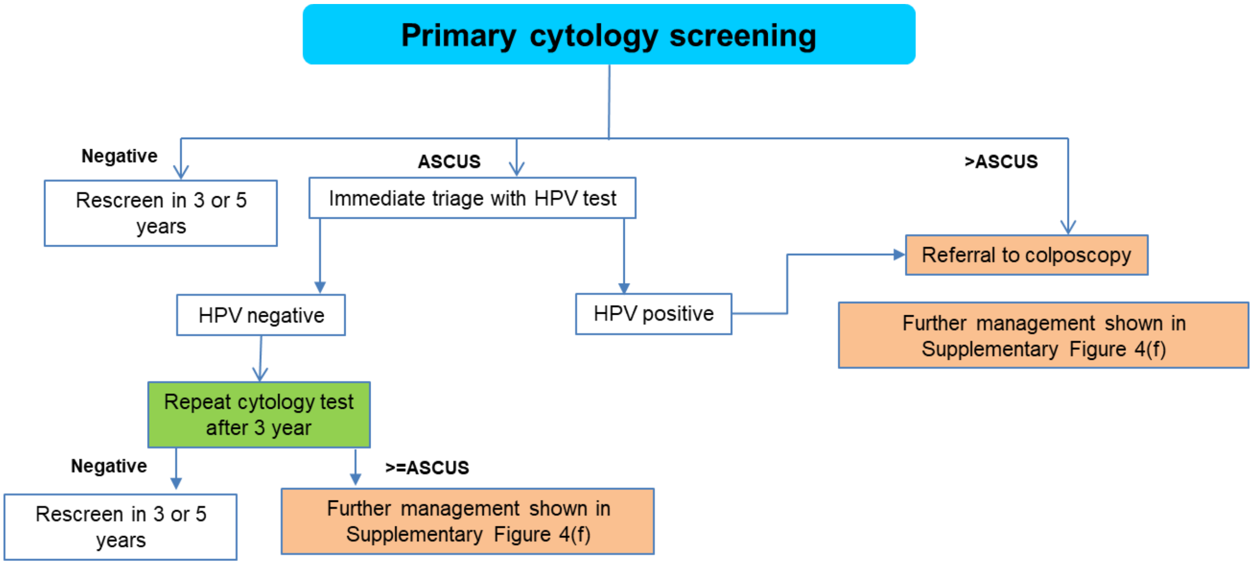

(c) Primary HPV without triage

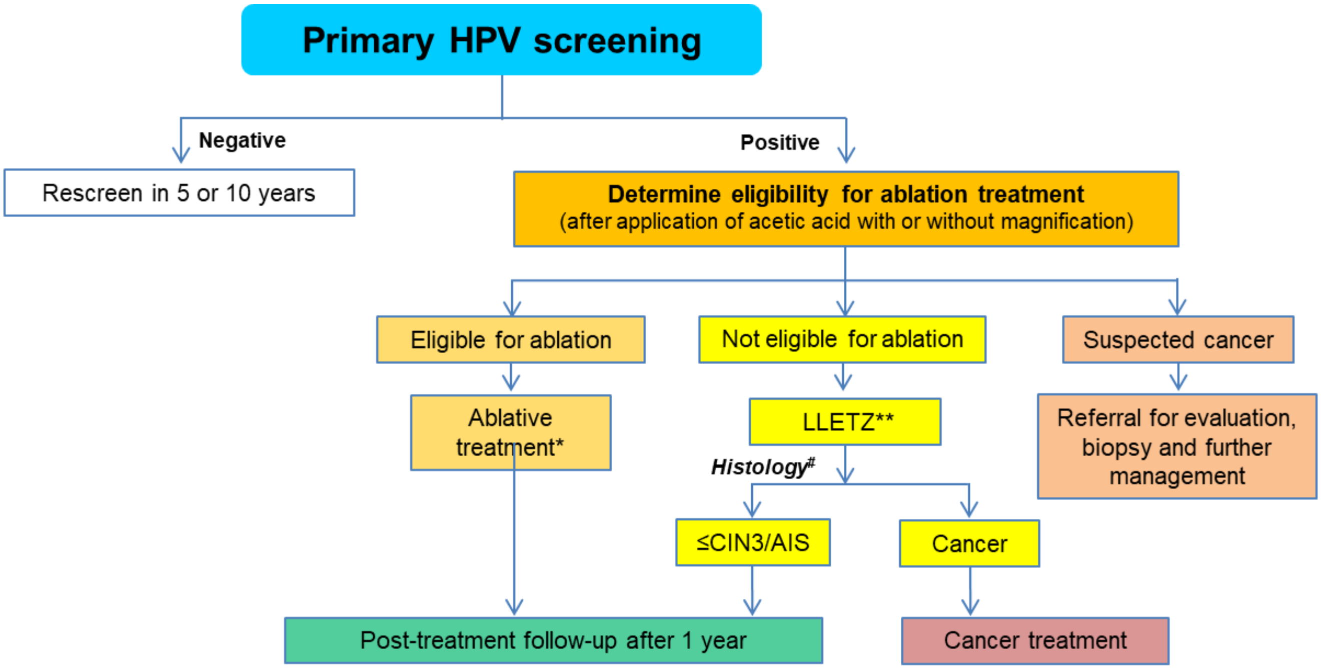

(d) Primary HPV, 16/18 triage

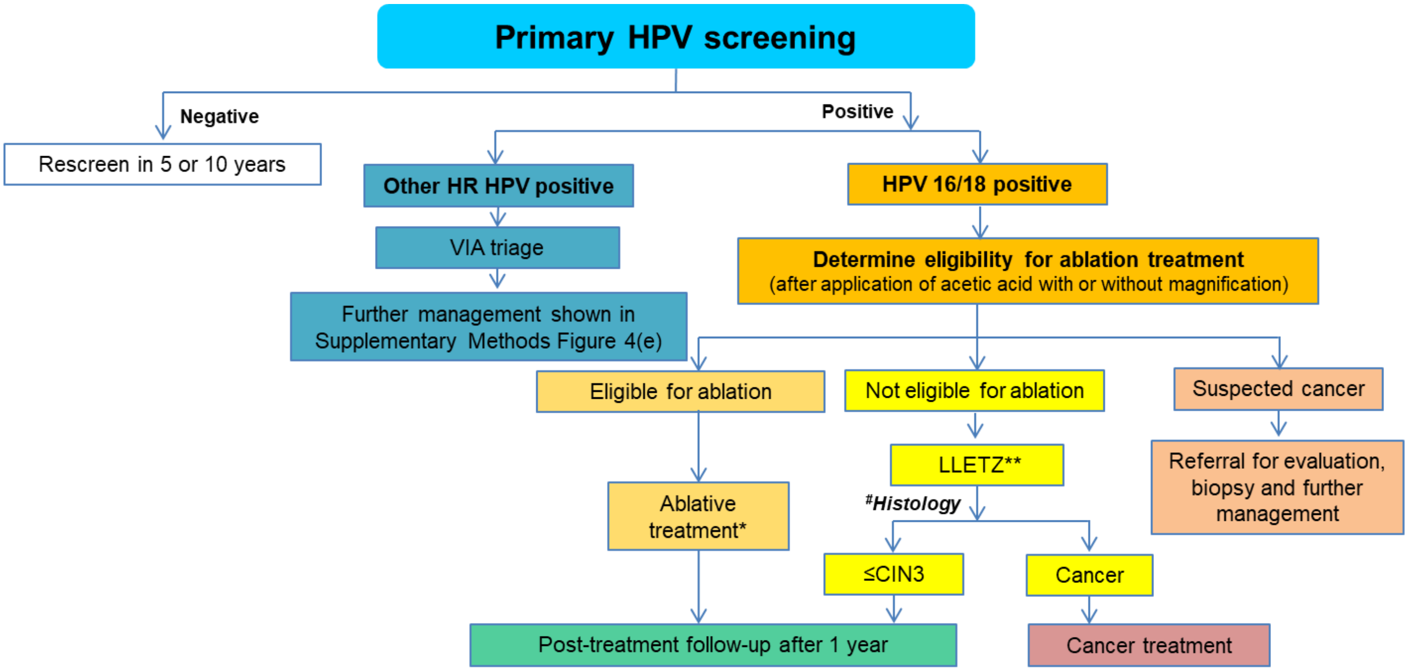

(e) Primary HPV, VIA triage

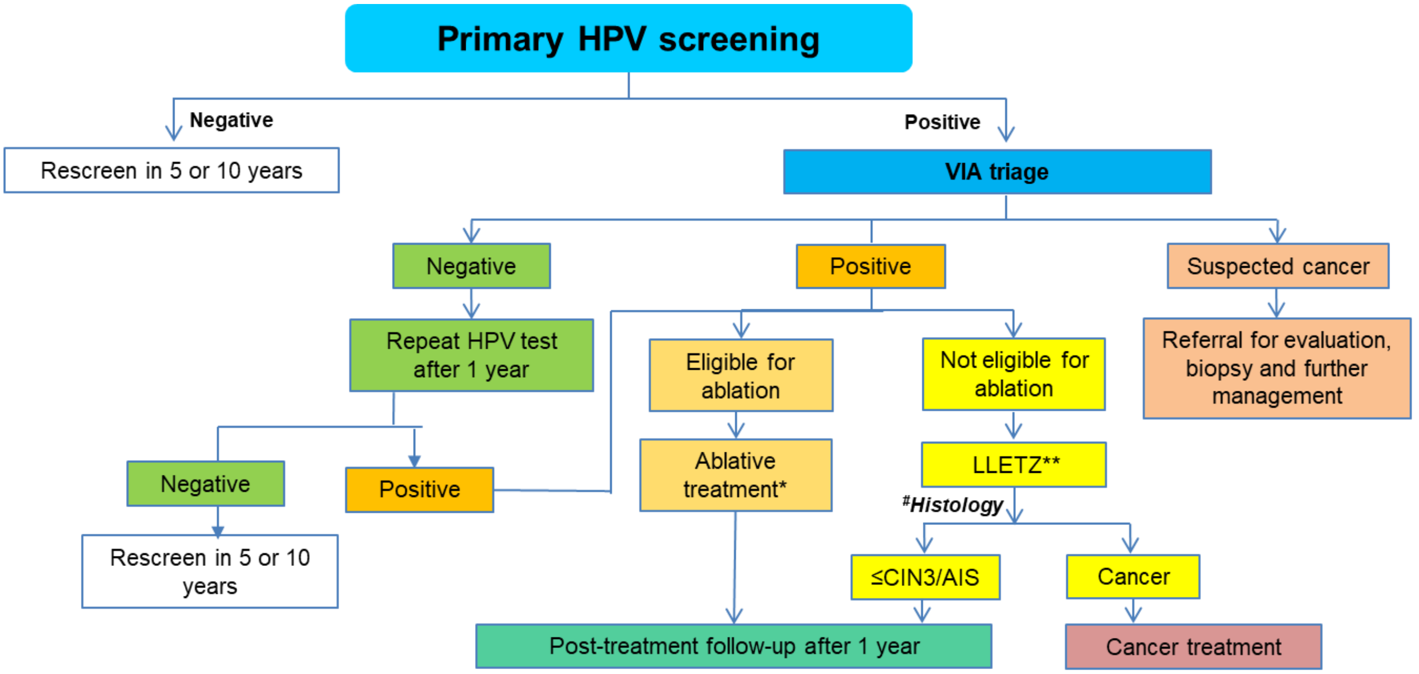

(f) Primary HPV, colposcopy triage^

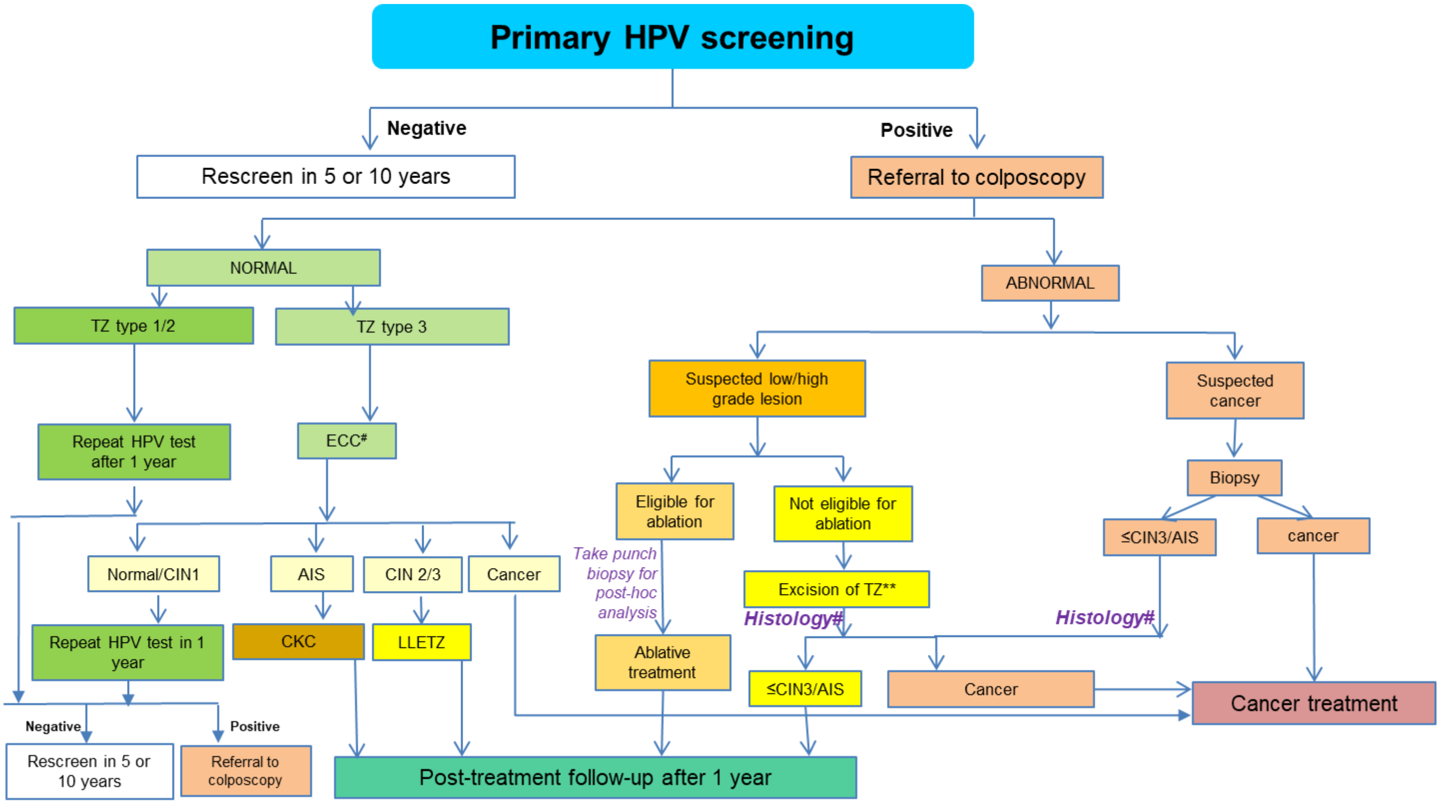

(g) Primary HPV (cytology triage)^

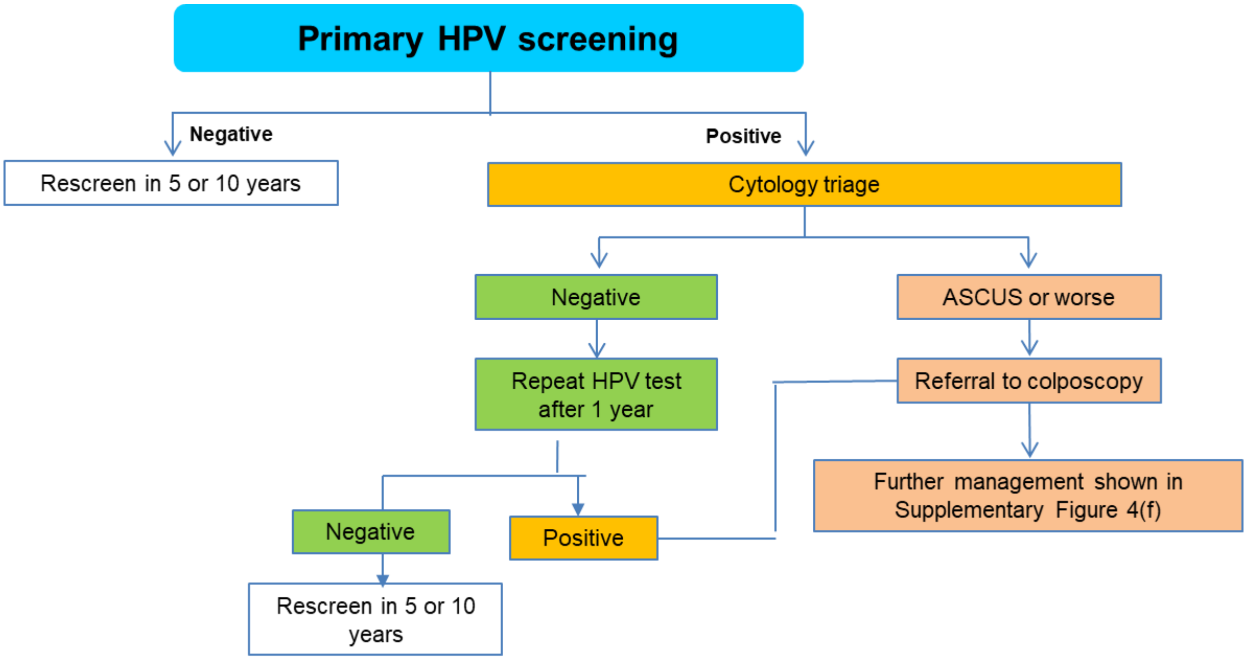

*Ablative treatment includes cryotherapy and thermal ablation. **CKC if LLETZ not available. # Histology may not be available in certain settings. Women should be followed-up after 1 year or to report earlier, if they have any of the symptoms of cervical cancer. ^ Women referred with cytology LSIL or worse at colposcopy will receive biopsy even if they have a type 1 or type 2 transformation zone at colposcopy.

Supplementary Table 1: Screening ages and frequencies considered for each screening algorithm

**Screening algorithms**	**Screening frequency and age-range (number of lifetime routine screening tests)**
**Primary VIA***	• 3 yearly, 30-50 years (7X)<br>• 5 yearly, 30-50 years (5X)
**Cytology, HPV triage****	
**Primary HPV***	• 5 yearly, 30-50 years (5X)<br>• 10 yearly, 30-50 years (3X)<br>• 10 yearly, 35-45 years (2X) 'Elimination strategy[$]'
**Primary HPV, HPV16/18 triage^**	
**Primary HPV, VIA triage^^**	
**Primary HPV, colposcopy triage**	
**Primary HPV, cytology triage****	

*All positive women treated after assessment of eligibility for ablative treatment. **Triage positive referred to colposcopy. ^^VIA triage positive women treated after assessment of eligibility for ablative treatment. ^HPV 16/18 positive women treated after assessment of eligibility for ablative treatment. Women positive for HPV types other than HPV 16/18 ('OHR') are triaged with VIA.
[$]The 'Elimination strategy' refers to the screening test, ages and frequencies assumed in the earlier analysis of cervical cancer elimination timeline.[9,15]

Supplementary Table 2: HPV-FRAME reporting standard checklist for the modelling in the general population of women

a) Inputs	Reported? (Y/N)	Reported by age? (Y/N)	Report by sex (F-only, M-only or both)?	Comments
**Core reporting standard**				
Target population for intervention	Y	Y	F	Cohort of women who would turn 30 in 2030 for the general population.<br>Screening algorithms target between ages 30-50.
Sexual behaviour	N	N	N	Implicitly accounted for in calibration. Data not directly used since vaccination is not modelled here and therefore a dynamic transmission model is not required.
Cohort examined for evaluation/ time horizon	Y	Y	F-only	The lifetime of a single cohort of women turning 30 in 2030. For CEA, discounting is applied from age 30 onwards.
Quality of life assumptions	Y	Y	F-only	Listed in Supplementary Table 8 (based on Global Burden of Disease study 2010[67]).
Calibration	Y	Y	F-only	Reproduces GLOBOCAN 2018 incidence at a country level. The models were then calibrated to final mortality outcomes to country- and age-specific rates from GLOBOCAN 2018 by incorporating a ‘quality factor’ into the final estimated country- and stage-specific survival assumptions. Details on this calibration in previous CCEMC work.[2,3]
Validation (where possible)	Y	Y (implicitly)	F-only	The models has previously been used to evaluate various HPV vaccination and cervical screening strategies for many countries, including high- resource countries, low-resource settings and globally (see Online Methods).

a) **Inputs**	**Reported? (Y/N)**	**Reported by age? (Y/N)**	**Report by sex (F-only, M-only or both)?**	**Comments**
Costs	Y	Y (implicitly)	F-only	Same unit costs are assumed regardless of age and are listed in Supplementary Table 2.
**Reporting standard for model of cervical screening**				
Routine screening behaviour (routine and follow-up and test of cure)	Y	Y	F-only	We assumed 70% of women attend a given routine screen with 90% adherence except for some circumstances such as where HPV point-of-care is possible. This is listed in the main text methods.
Screening test (s) and colposcopy accuracies	Y	Y (implicitly)	F-only	Sensitivity and specificity of tests for CIN2+ are listed in Supplementary Tables 3-4.
Abnormal test management (primary and triage)	Y	Y	F-only	Flowcharts are detailed in Supplementary Figure 4 and the algorithms (age eligibility, routine screening frequency) are detailed in Supplementary Table 1.
Diagnostic follow-up of abnormal tests	Y	Y	F-only	Flowcharts are detailed in Supplementary Figure 4
Management by disease grade (confirmed disease)	Y	Y	F-only	Flowcharts are detailed in Supplementary Figure 4
Sources of information for screening structure and parameterization	Y	Y	F-only	Specified in main text and Online Methods as being informed by the Guidelines Development Group.
**Reporting standard for models of HPV prevention in LMIC**				
HIV prevalence rates, if endemic in country	N	N	N	We did not explicitly take into account HIV prevalence in this study. This is addressed in a companion study.
Description of any opportunistic or pilot/demonstration	N	N	N	As this study models the impact of HPV vaccination and cervical screening strategies in 78 LMICs, this is not relevant.

a) Inputs	Reported? (Y/N)	Reported by age? (Y/N)	Report by sex (F-only, M-only or both)?	Comments
screening project ongoing				

b) Outputs	Reported? (Y/N)	Reported by age? (Y/N)	Report by sex (F-only, M-only or both)?	Report as calibration or validation target? (Y/N)
**Core reporting standard**				
Cancer incidence, mortality, life years, QALYs/DALYs (as appropriate)	Y	Y (implicitly)	F-only	Age-standardized and age-specific incidence and mortality rates were reported along with cases and deaths per 100,000 women. HALYS (Health-adjusted life years saved) were reported for the main results and life years were reported as part of the sensitivity analysis. Age-specific results not directly reported but are critical to calculation of HALYS and ASRs.
HPV prevalence, pre-intervention	N	N	N	This level of detail is not reported. Herd immunity effects could be a factor for vaccination but vaccination is not modelled in this paper.
CIN2 detected	N	N	N	This level of detail is not reported.
Sensitivity analysis on key inputs	Y	Y (implicitly)	F-only	One-way sensitivity analysis were performed for adherence rates and primary test performance. Life years were assessed along with equal discounting for both HALYs and costs. Probabilistic sensitivity analysis was performed for costs.
Incremental cost-effectiveness ratios and costs saved	Y	N	F-only	ICERs along the cost-effectiveness frontiers are displayed in the main text and Extended Display Items (for sensitivity analysis)

QALYs: quality-adjusted life-years

Supplementary Table 3: Cross-sectional sensitivity and specificity inputs used for (a) primary test technologies and (b) combined primary and triage test outputs. We also present assumptions for test performance considered in sensitivity analysis for primary test technologies in (c)

(a)

**Primary test**	**Sensitivity CIN2+ targets**	**Specificity CIN2+ targets**	**Model calculated sensitivity to CIN2+[@]**	**Model calculated specificity to CIN2+[@]**
**Primary VIA**	41% CIN2+ as a realistic sensitivity.<br>Additionally present a high sensitivity scenario (60% CIN2+) for all outputs.	78%	39.1-42.8%	78.0-80.8%
**Primary cytology (LSIL cut-off)**	LBC: 70.3% (59.7-79.1%)<br>Conv: 62.8% (46.8-76.5%)	LBC: 96.2% (94.6-97.4%)<br>Conv: 97.7% (96.1-98.7%)	67-71.3%	94.1-97.8%
**Primary HPV**	92.60% (89.25-95.30%)	89.30% (87.03%-91.20%)	96.6-98.1%	73.0-97.0%

(b)

**Primary/triage test combinations**	**Sensitivity CIN2+ targets**	**Specificity CIN2+ targets as**	**Model calculated sensitivity to CIN2+[@]**	**Model calculated specificity to CIN2+[@]**

**HPV positive, HPV 16/18 triage***	52.9% (95% CI 50.2-55.7%)	74.5% (95% CI 70.3-79.0%)	46.3-75.9%	67.2-86.3%
**HPV positive, Cytology triage (ASCUS)***	71.5% (95%CI: 65.2-77.1%)	74.7% (95% CI 69.2-79.5%)	70.1-74.7%	68.1-85.2%
**HPV positive, VIA triage ***	Range: 45.2% to 84.2%	Range: 44.8% to 94.5%	39.2-42.99%	73.3-76.9%
**Primary cytology (LSIL cut-off)**	LBC: 70.3% (59.7-79.1%) Conv: 62.8% (46.8-76.5%)	LBC: 96.2% (94.6-97.4%) Conv: 97.7% (96.1-98.7%)	67-71.3% Sensitivity analysis**: 46.8% (lower bound) – 80% (upper bound)	94.1-97.8%
**Primary HPV**	92.60% (89.25-95.30%)	89.30% (87.03%-91.20%)	96.6-98.1% Sensitivity analysis**: 88% (lower bound) – 95.7% (upper bound)	73.0-97.0%
**Cytology ASCUS, HPV triage**	96.2% (95% CI : 91.7–98.3%)	54.9% (95% CI:43.5–65.9%)	96.9-98.2%	39.2-88.1%
**Primary VIA**	41% Range: 30-60%	78%	Lower bound: 39.1-42.8% Upper bound: 60-64%	Lower bound: 78.0-80.8% Upper bound: 78.6-79.4%

*Data available for women aged 35-60. ^Liquid-based cytology assumed, and HPV test using ATIMA.**Sensitivity analysis on the sensitivity of primary HPV, primary cytology and primary VIA are performed on predictions of incidence and mortality reductions. @Range of sensitivity and specificity here. We assume a fixed test performance for each test across all scenarios. As disease burden varies substantially across all-78 LMIC, equivalent test performance may result in different sensitivity and specificity at a population-level; for instance, tests used in settings with high rates of CIN2/3 will generate different sensitivity calculation compared with settings with very low rates of disease.

(c)

Primary test	Sensitivity CIN2+ data
**Primary VIA**	Based on combined evidence from systematic reviews and population longitudinal data we consider range of 30-60^%
**Primary cytology (LSIL cut-off)**	Based on performance across conventional and LBC, we consider range of 46.8%-80%
**Primary HPV**	Based on performance reported from studies, we consider range of 88%-96%

^60% sensitivity values are presented explicitly for all scenarios.

Supplementary Table 4: Probability of receiving a specific test result by underlying health state for (a) VIA, (b) VIA (high sensitivity), (c) VAT, (d) cytology, (e) HPV. The same test probability matrix is used when the test is used as a primary or triage test.

(a) VIA

Health state	Negative	Positive (eligible for ablation)	Positive (ineligible for ablation)	Suspicious for cancer
**Well**	81.0%	18.05%	0.95%	0%
**HPV (no CIN)**	75.0%	23.75%	1.25%	0%
**Productive HPV infection (CIN1)**	70.0%	27.0%	3.0%	0%
**CIN2**	65.0%	24.5%	10.5%	0%
**CIN3**	59.0%	20.5%	16.4%	0%
**Cervical cancer**	40.0%	0%	0%	60.0%

(b) VIA (high sensitivity)

Health state	Negative	Positive (eligible for ablation)	Positive (ineligible for ablation)	Suspicious for cancer
**Well**	80.0%	19.0%	1.0%	0%
**HPV (no CIN)**	74.0%	24.7%	1.3%	0%
**Productive HPV infection (CIN1)**	69.0%	27.9%	3.1%	0%
**CIN2**	50.0%	35.0%	15.0%	0%
**CIN3**	37.0%	31.5%	25.2%	6.3%
**Cervical cancer**	12.0%	0%	0%	88.0%

(c) VIA used as a visual assessment for treatment

Health state	Eligible for ablation	Ineligible for ablation
**Well**	99.05%	0.95%
**HPV (no CIN)**	98.75%	1.25%
**Productive HPV infection (CIN1)**	97.0%	3.0%
**CIN2**	89.50%	10.5%
**CIN3**	79.5%	16.4%
**Cervical cancer**	40.0%	60.0%

(d) Cytology

Health state	Negative	ASC-US	LSIL	ASC-H+
**Well**	96.72%	1.35%	1.33%	0.6%
**HPV (no CIN)**	89.3%	4.8%	4.8%	1.1%
**Productive HPV infection (CIN1)**	45.95%	24.69%	20.58%	8.78%
**CIN2**	41.59%	1.71%	24.3%	32.4%
**CIN3**	24.49%	3.51%	25.2%	46.8%

(e) HPV

Health state	Negative	Positive
**Well**	98.6%	1.4%
**HPV (no CIN)**	44.0%	56.0%
**Productive HPV infection (CIN1)**	15.85%	84.15%
**CIN2**	7.0%	93.0%
**CIN3+**	1.6%	98.40%

Supplementary Table 5: List of included LMIC in the analysis

Country	WHO Region	Female population, 2020 (30-49 years) ('000)*	GDP per-capita (World Bank 2019 unless noted otherwise)
Afghanistan	South Asia	3586	507
Angola	Sub-Saharan Africa	3138	2791
Bangladesh	South Asia	23841	1856
Benin	Sub-Saharan Africa	1222	1219
Bhutan	South Asia	103	3316
Bolivia	Latin America & Caribbean	1437	3552
Burkina Faso	Sub-Saharan Africa	2029	787
Burundi	Sub-Saharan Africa	1572	261
Cape Verde	Sub-Saharan Africa	77	3604
Cambodia	East Asia & Pacific	2246	1643

Country	WHO Region	Female population, 2020 (30-49 years) ('000)*	GDP per-capita (World Bank 2019 unless noted otherwise)
Cameroon	Sub-Saharan Africa	2770	1507
Central African Republic	Sub-Saharan Africa	423	468
Chad	Sub-Saharan Africa	1424	710
Comoros	Sub-Saharan Africa	96	1370
Congo Dem. Rep.	Sub-Saharan Africa	8248	581
Congo	Sub-Saharan Africa	608	2280
Cote d'Ivoire	Sub-Saharan Africa	2625	2276
Djibouti	Middle East & North Africa	135	3415
Egypt	Middle East & North Africa	12972	3019
El Salvador	Latin America & Caribbean	903	4187
Eritrea	Sub-Saharan Africa	374	567*
Ethiopia	Sub-Saharan Africa	11462	856
The Gambia	Sub-Saharan Africa	237	778
Georgia	Europe & Central Asia	553	4698
Ghana	Sub-Saharan Africa	3555	2202
Guinea	Sub-Saharan Africa	1298	963
Guinea-Bissau	Sub-Saharan Africa	215	697
Haiti	Latin America & Caribbean	1460	1272
Honduras	Latin America & Caribbean	1270	2575

Country	WHO Region	Female population, 2020 (30-49 years) ('000)*	GDP per-capita (World Bank 2019 unless noted otherwise)
India	South Asia	185040	2100
Indonesia	East Asia & Pacific	39405	4136
Kenya	Sub-Saharan Africa	6259	1817
Korea Dem. Rep.	East Asia & Pacific	3644	640*
Kyrgyz Republic	Europe & Central Asia	858	1309
Lao PDR	East Asia & Pacific	927	2535
Lesotho	Sub-Saharan Africa	258	1118
Liberia	Sub-Saharan Africa	535	622
Madagascar	Sub-Saharan Africa	2889	523
Malawi	Sub-Saharan Africa	1912	412
Mali	Sub-Saharan Africa	1839	879
Mauritania	Sub-Saharan Africa	517	1679
Moldova	Europe & Central Asia	668	4504
Mongolia	East Asia & Pacific	497	4340
Morocco	Middle East & North Africa	5232	3204
Mozambique	Sub-Saharan Africa	3094	504
Myanmar	East Asia & Pacific	7926	1408
Nepal	South Asia	4230	1071
Nicaragua	Latin America & Caribbean	942	1913
Niger	Sub-Saharan Africa	1978	554
Nigeria	Sub-Saharan Africa	20453	2230

Country	WHO Region	Female population, 2020 (30-49 years) ('000)*	GDP per-capita (World Bank 2019 unless noted otherwise)
Pakistan	South Asia	25495	1285
Papua New Guinea	East Asia & Pacific	1058	2829
Philippines	East Asia & Pacific	13821	3485
Rwanda	Sub-Saharan Africa	1498	820
Sao Tome and Principe	Sub-Saharan Africa	23	1947
Senegal	Sub-Saharan Africa	1819	1447
Sierra Leone	Sub-Saharan Africa	832	528
Solomon Islands	East Asia & Pacific	77	2374
Somalia	Sub-Saharan Africa	1321	105*
South Sudan	Sub-Saharan Africa	1119	1120 (2015)
Sri Lanka	South Asia	3006	3853
Sudan	Sub-Saharan Africa	4560	442
Swaziland	Sub-Saharan Africa	174	3895
Syrian Arab Republic	Middle East & North Africa	2440	1194*
Tajikistan	Europe & Central Asia	1144	871
Tanzania	Sub-Saharan Africa	6009	1122
Timor-Leste	East Asia & Pacific	129	1561
Togo	Sub-Saharan Africa	911	679
Tunisia	Middle East & North Africa	1792	3317
Uganda	Sub-Saharan Africa	4283	794
Ukraine	Europe & Central Asia	6861	3659

**Country**	**WHO Region**	**Female population, 2020 (30-49 years) ('000)***	**GDP per-capita (World Bank 2019 unless noted otherwise)**
Uzbekistan	Europe & Central Asia	4791	1725
Vanuatu	East Asia & Pacific	36	3115
Viet Nam	East Asia & Pacific	14787	2715
Palestine	Middle East & North Africa	548	3562 (2018)
Yemen	Middle East & North Africa	3217	774
Zambia	Sub-Saharan Africa	1838	1305
Zimbabwe	Sub-Saharan Africa	1727	1464

* Sourced from UN Data. 2019. (Accessed February 10, 2021 at https://data.un.org/)

Supplementary Table 6: Aggregate costs across 78 LMICs for each screening-related event.[+]

Event	Cost (US$ 2019)	
	**Base-case**	**Range in sensitivity analysis**
**Primary VIA^**	7.12	+/-20% (5.70-8.54)
**Primary HPV DNA (+/-16/18)***	15.09	+/-30% (10.56-19.62)
**Primary cytology^**	18.02	+/-20% (14.42-21.62)
**VIA triage**[O]	2.95	+/-20% (2.36-3.54)
**Cytology triage**[O]	15.62	+/-20% (12.5-18.74)
**HPV triage**[O]	8.15	Upper end informed by current high-end values; lower end represents potential cost at higher volumes (5-10.06)
**Colposcopy**[O,#]	9.96	-
**Ablative treatment**	11.76	+/-30% (8.23-15.29)
**Excisional treatment**	41.67	+/-30% (29.17-54.17)
**Histology**[@]	17.96	-
**Punch biopsy/Biopsy**	11.61	-
**Endocervical curettage (ECC)**	6.4	-
**Cancer diagnosis and treatment– FIGO 1**[a]	261.43	one-way: +40%, no lower bound (366.00)<br>For PSA:+/-20% (209.14-313.72)
**Cancer diagnosis and treatment– FIGO 2**[a]	540.23	one-way: +40%, no lower bound (756.32)<br>For PSA:+/-20% (432.18-648.28)
**Cancer diagnosis and treatment– FIGO 3**[a]	673.93	one-way: +40%, no lower bound (943.50)<br>For PSA:+/-20% (539.14-808.72)
**Cancer diagnosis and treatment– FIGO 4**[a]	307.95	one-way: +40%, no lower bound (431.13)<br>For PSA:+/-20% (246.36-369.54)
**Palliative care**[a]	115.13	-
**Yearly surveillance after treatment**[a]	57.66	-

+Aggregate costs represent the average across 78 LMIC, i.e. the sum of the country-level costs weighted by the proportion of the 78 LMIC population of 30-49 year-old females in each country.
^ Includes consumables, administering provider/workforce, and programmatic utilisation costs.
* Includes cost of test, sample drop-off and transport, laboratory staff time, lab supplies, general administration and overhead costs of primary screening using WHO-CHOICE methodology and database.
[O] Same as primary, but excludes a proportion of the labour, programmatic and utilisation costs from primary visits due to not requiring another visit. When VIA is used during colposcopy, we assume no cost.
Includes consumables/equipment, workforce.
@Includes consumables/equipment, workforce including pathologist and biomedical scientist.
[a]Cancer costs are only applied to the proportion of cancers that are treated and assumed to apply to 90% of screen-detected cases in the base case. Surveillance costs are applied from 1 year after diagnosis until death, or a maximum of 5 years if the woman survives for this amount of time.

Supplementary Table 7: Aggregate costs across each region for each screening-related event.[+]

Event	Cost (US$ 2019)					
	East Asia & Pacific	Europe & Central Asia	Latin America & Caribbean	Middle East & North Africa	South Asia	Sub-Saharan Africa
**Primary VIA^**	7.39	15.59	7.83	8.82	5.71	7.77
**Primary HPV DNA (+/- 16/18)***	14.07	21.42	16.47	17.16	14.01	16.46
**Primary cytology^**	16.9	37.08	19.86	21.97	15.55	19.46
**VIA triage[O]**	2.01	8.89	4.55	3.25	2.23	4.23
**Cytology triage[O]**	14.25	31.24	17.44	19.04	13.57	17.19
**HPV triage[O]**	8.15	8.15	8.15	8.15	8.15	8.15
**Colposcopy[O,#]**	9.92	20.12	10.8	11.73	8.57	10.47
**Ablative treatment**	11.71	22.32	12.53	13.31	10.5	11.97
**Excisional treatment**	41.58	53.41	43.55	43.72	40.03	42.36
**Histology[@]**						
**Punch biopsy/Biopsy**	15.8	32.09	20.4	21.52	16.14	19.9
**ECC**	11.22	23	12.9	14.04	9.9	12.68
**Cancer diagnosis and treatment– FIGO 1[a]**	4.94	11.66	7.47	8.31	5.68	7.58
**Cancer diagnosis and treatment– FIGO 2[a]**	241.24	329.07	327.26	307.84	262.29	277.57
**Cancer diagnosis and treatment– FIGO 3[a]**	463.28	648.75	670.03	571.68	555.75	611.42
**Cancer diagnosis and treatment– FIGO 4[a]**	555.59	813.31	850.77	695.41	699.96	785.4
**Palliative care[a]**	244.31	367.05	389.39	303.95	323.07	369.26
**Yearly surveillance after treatment[a]**	48.89	69.88	72.42	61.11	59.47	65.75

+Aggregate costs represent the average across 78 LMIC, i.e. the sum of the country-level costs weighted by the proportion of the 78 LMIC population of 30-49 year-old females in each country.
^ Includes consumables, administering provider/workforce, and programmatic utilisation costs.
* Includes cost of test, sample drop-off and transport, laboratory staff time, lab supplies, general administration and overhead costs of primary screening using WHO-CHOICE methodology and database.
[O] Same as primary, but excludes a proportion of the labour, programmatic and utilisation costs from primary visits due to not requiring another visit. When VIA is used during colposcopy, we assume no cost.
Includes consumables/equipment, workforce.
@Includes consumables/equipment, workforce including pathologist and biomedical scientist.
[a]Cancer costs are only applied to the proportion of cancers that are treated and assumed to apply to 90% of screen-detected cases in the base case. Surveillance costs are applied from 1 year after diagnosis until death, or a maximum of 5 years if the woman survives for this amount of time.

Supplementary Table 8: Aggregate disutilities across 78 LMICs+

Event	Annualized disutility weight		Duration disutility is applied
	**Baseline**	**Range in sensitivity analysis**	
**Cancer diagnosis and treatment– FIGO 1[a]**	0.288	For PSA: 0-100% (0-0.288)*	Year of diagnosis
**Cancer diagnosis and treatment– FIGO 2[a]**	0.288	For PSA: 0-100% (0-0.288)*	Year of diagnosis
**Cancer diagnosis and treatment– FIGO 3[a]**	0.288	For PSA: 0-100% (0-0.288)*	Year of diagnosis
**Cancer diagnosis and treatment– FIGO 4[a]**	0.451	For PSA: 0-100% (0-0.451)*	Year of diagnosis until last 3 months of life
**Palliative care[a]**	0.54	For PSA: 0-100% (0-0.54)*	Last 3 months of life
**Yearly surveillance after treatment[a,b]**	0.049	For PSA: 0-100% (0-0.049)*	Every year of cancer after the first year up to the last 3 months of life (or survival)
**Pre-cancer treatment**	0	0.01 assumed for one-way sensitivity analysis.	(Sensitivity analysis): year of pre-cancer treatment

+Aggregate costs represent the average across 78 LMIC, i.e. the sum of the country-level costs weighted by the proportion of the 78 LMIC population of 30-49 year-old females in each country.
* All disutilities are varied together within PSA, i.e. all events would have a value of X% multiplied by their baseline. This is to avoid unrealistic scenarios that could occur if they varied independently such as the FIGO1 diagnosis and treatment having a higher disutility than FIGO4 diagnosis and treatment.
[a] Cancer disutilities are only applied to the proportion of cancers that are treated and assumed to apply to 90% of screen-detected cases. Surveillance disutilities are applied from 1 year after diagnosis until death, or a maximum of 5 years if the woman survives for this amount of time.
[b] Excludes FIGO4
