# Supplementary material for: Benefits, harms and cost-effectiveness of cervical screening, triage and treatment strategies for women in the general population
Source: Nat Med. 2023 Dec 12;29(12):3050–8. doi: 10.1038/s41591-023-02600-4 (PMC10719104; doi:10.1038/s41591-023-02600-4)
Supplement: Supplementary file 2 — Reporting Summary [file 41591_2023_2600_MOESM2_ESM.pdf]

nature portfolio

Corresponding author(s): Kate Simms and Karen Canfell

Last updated by author(s): May 25, 2023

Reporting Summary

Nature Portfolio wishes to improve the reproducibility of the work that we publish. This form provides structure for consistency and transparency in reporting. For further information on Nature Portfolio policies, see our Editorial Policies and the Editorial Policy Checklist.

Statistics

For all statistical analyses, confirm that the following items are present in the figure legend, table legend, main text, or Methods section.

n/a	Confirmed	
☐	☒	The exact sample size (*n*) for each experimental group/condition, given as a discrete number and unit of measurement
☒	☐	A statement on whether measurements were taken from distinct samples or whether the same sample was measured repeatedly
☒	☐	The statistical test(s) used AND whether they are one- or two-sided<br>*Only common tests should be described solely by name; describe more complex techniques in the Methods section.*
☒	☐	A description of all covariates tested
☒	☐	A description of any assumptions or corrections, such as tests of normality and adjustment for multiple comparisons
☐	☒	A full description of the statistical parameters including central tendency (e.g. means) or other basic estimates (e.g. regression coefficient) AND variation (e.g. standard deviation) or associated estimates of uncertainty (e.g. confidence intervals)
☒	☐	For null hypothesis testing, the test statistic (e.g. *F*, *t*, *r*) with confidence intervals, effect sizes, degrees of freedom and *P* value noted<br>*Give P values as exact values whenever suitable.*
☒	☐	For Bayesian analysis, information on the choice of priors and Markov chain Monte Carlo settings
☒	☐	For hierarchical and complex designs, identification of the appropriate level for tests and full reporting of outcomes
☒	☐	Estimates of effect sizes (e.g. Cohen's *d*, Pearson's *r*), indicating how they were calculated

Our web collection on statistics for biologists contains articles on many of the points above.

Software and code

Policy information about availability of computer code

Data collection	We used publicly available data for this evaluation. Any additional data that was provided is described in the Online Methods. No software was required for data collection.
Data analysis	No software was required to analyse the data that was provided.

For manuscripts utilizing custom algorithms or software that are central to the research but not yet described in published literature, software must be made available to editors and reviewers. We strongly encourage code deposition in a community repository (e.g. GitHub). See the Nature Portfolio guidelines for submitting code & software for further information.

Data

Policy information about availability of data

All manuscripts must include a data availability statement. This statement should provide the following information, where applicable:

- Accession codes, unique identifiers, or web links for publicly available datasets
- A description of any restrictions on data availability
- For clinical datasets or third party data, please ensure that the statement adheres to our policy

Data on cost inputs (provided separately for the six regions), test performance, health economic parameters, screening algorithms and compliance assumptions relevant to this specific evaluation are described in the online methods section. Data on country-specific GDP per-capita is also provided in the online methods.

Demographic data and data informing the calibration to cervical cancer incidence and mortality across the 78-LMICs and separately for the six regions is described in the Online Methods and in more detail in earlier publications9,13.

Human research participants

Policy information about studies involving human research participants and Sex and Gender in Research.

Reporting on sex and gender	Not applicable. This is a modelling study
Population characteristics	Not applicable. This is a modelling study
Recruitment	Not applicable. This is a modelling study
Ethics oversight	Not applicable. This is a modelling study

Note that full information on the approval of the study protocol must also be provided in the manuscript.

Field-specific reporting

Please select the one below that is the best fit for your research. If you are not sure, read the appropriate sections before making your selection.

☒ Life sciences ☐ Behavioural & social sciences ☐ Ecological, evolutionary & environmental sciences

For a reference copy of the document with all sections, see nature.com/documents/nr-reporting-summary-flat.pdf

Life sciences study design

All studies must disclose on these points even when the disclosure is negative.

Sample size	We simulated thirty million women from birth to age 85 years across 78-LMICs for each of the six regions we modelled, as described in our methods. We chose this many women to ensure that the outcomes for life-years, cancer incidence and mortality were smooth and that randomness in the simulation did not impact outputs.
Data exclusions	No data were excluded.
Replication	The model simulation required no replication. By choosing a large enough number of women to simulate, we were ensured that randomness in the model runs would not impact the results of the simulation.
Randomization	This is a modelling study, In the model simulation, random numbers are generated throughout when evaluating probabilities that women experience certain key events e.g contract an HPV infection.
Blinding	this is a modelling study and blinding is not required.

Reporting for specific materials, systems and methods

We require information from authors about some types of materials, experimental systems and methods used in many studies. Here, indicate whether each material, system or method listed is relevant to your study. If you are not sure if a list item applies to your research, read the appropriate section before selecting a response.

Materials & experimental systems

n/a	Involved in the study	
☒	☐	Antibodies
☒	☐	Eukaryotic cell lines
☒	☐	Palaeontology and archaeology
☒	☐	Animals and other organisms
☒	☐	Clinical data
☒	☐	Dual use research of concern

Methods

n/a	Involved in the study	
☒	☐	ChIP-seq
☒	☐	Flow cytometry
☒	☐	MRI-based neuroimaging
